# The Infant Health Study - Promoting mental health and healthy weight through sensitive parenting to infants with cognitive, emotional, and regulatory vulnerabilities: protocol for a stepped-wedge cluster-randomized trial and a process evaluation within municipality settings

**DOI:** 10.1186/s12889-022-12551-z

**Published:** 2022-01-28

**Authors:** Anne Mette Skovgaard, Marian Bakermans-Kranenburg, Maiken Pontoppidan, Tine Tjørnhøj-Thomsen, Katrine Rich Madsen, Ida Voss, Stine Kjær Wehner, Trine Pagh Pedersen, Lotte Finseth, Rodney S. Taylor, Janne Schurmann Tolstrup, Janni Ammitzbøll

**Affiliations:** 1 National Institute of Public Health, NIPH, University of Southern Denmark, Copenhagen, Denmark; 2 Clinical Child and Family Studies, Vrije Universiteit, Amsterdam, The Netherlands; 3 The Danish Center for Social Science Research, VIVE, Copenhagen, Denmark; 4 MRC/CSO Social and Public Health Sciences Unit & Robertson Centre for Biostatistics, Institute of Health and Well Being, University of Glasgow, Glasgow, UK; 5 University of Exeter, Exeter, United Kingdom

**Keywords:** Infants, Mental health problems, Unhealthy weight, Early intervention, Community health services, Sensitive parenting, Step-wedge randomized controlled trial, Mixed-methods, Process-evaluation

## Abstract

**Background:**

Child mental health problems are a major public health concern associated with poor mental and physical health later in development. The study evaluates a new community-based intervention to promote sensitive parenting and reduce enduring mental health problems and unhealthy weight among vulnerable infants aged 9-24 months.

**Methods:**

We use a step-wedge cluster randomized controlled trial design conducted within a home visiting program offered by community health nurses to infant families in Denmark. Sixteen municipalities are randomly allocated to implement the intervention starting at three successive time points from May 1, 2022 to January 1, 2023. A total of 900-1000 families will be included. A standardized program, Psykisk Udvikling og Funktion (PUF), is used to identify infants with major problems of eating, sleep, emotional or behavioral regulation or developmental problems. The intervention builds on the Video-Feedback Intervention to Promote Positive Parenting (VIPP) program, adapted to the PUF-context and named the VIPP-PUF. Children will be followed up at ages 18 and 24 months. Primary outcome measure is the Strengths and Difficulties Questionnaire (SDQ) at child age 24 months. The other outcome measures include body mass index z-scores, the Ages and Stages Questionnaire Social-Emotional (ASQ:SE2); the Child Behavior Checklist (CBCL 1½ -5); Eating behavior Questionnaires; the Being a Mother-questionnaire (BaM13); the Parental Stress Scale (PSS); and the WHO-5 well-being index (WHO-5). Data on child and family factors are obtained from National registries and the Child Health Database.

Quantitative measures are applied to examine the effectiveness of the VIPP-PUF intervention and the implementation process. Qualitative measures include interviews with CHNs, parents and municipality stakeholders to explore factors that may influence the adherence and effectiveness of the intervention.

**Discussion:**

The study examines a service-setting based intervention building on the promotion of sensitive parenting to vulnerable infants. We use a mixed methods approach to evaluate the intervention, taking into account the influences of COVID-19 pandemic running since March 2020. Overall, the study has potential to add to the knowledge on the possibilities of prevention within the municipality child health care to reduce the risk of mental health problems and unhealthy weight in early childhood.

**Trial registration:**

www.ClinicalTrials.gov; IDNCT04601779; Protocol ID 95-110-21307. Registered 25 June 2021.

**Supplementary Information:**

The online version contains supplementary material available at 10.1186/s12889-022-12551-z.

## Background

Childhood mental health problems are highly prevalent, and they have severe long-term prognoses regarding mental and physical health in older ages [1–3]. The overall population prevalence of mental health problems across the range of neuro-developmental, emotional, and behavioral problems is 10-20% [4–6] with higher estimates seen in socially deprived populations and among children from families of psycho-social disadvantage, particularly families with young parents, single parents, parents of low education, and mentally ill parents [7–10].

Mental disorders of neurodevelopmental origin such as intellectual disability, autism spectrum disorders and disorders of hyperactivity and inattention, ADHD, are characterized by an onset in early childhood, and increasing research evidence indicate that problems of motor and language development, inattention, contact and communication in infancy are reliable predictors of these disorders in preschool to school age [11–16]. Also, infancy regulation problems involving eating, sleep, and emotional and behavioral reactions have been found predictive of mental health problems and disorders later in preschool to school age [16–18]. Also, developmental trajectories of dysregulation in infancy have been found associated with an up to tenfold increased risk of mental health problems later in childhood, particularly ADHD, eating problems, and emotional- and behavioral problems [17–22].

The relation between the parents and the child has overarching importance for the child’s development. Thus, parenting behavior may have protective as well as negative impact on the developmental trajectories of the child’s mental and physical health [23–25], and parenting plays a key role in the maintenance of enduring problems in vulnerable infants [20, 26–30].

### Associations between mental health and healthy weight in childhood

Co-occurrence of mental health problems and overweight has repeatedly been found among older children and adolescents [31–37], and longitudinal data indicate that mental health problems tend to precede the development of overweight [34–36, 38]. The risk trajectories of unhealthy weight development are far from understood, but mounting evidence suggests a highly increased risk of overweight in school ages in children who were overweight in infancy [39–44]. The identified risk factors of childhood overweight include young parental age, low maternal education, parents who are overweight or obese, single parent families, and ethnic minorities [45–47]. Among Danish children however, a more than sevenfold increased risk of overweight at ages 5-8 years has been found in children who are overweight and obese in infancy, independent of child and family factors [48]. Overall, there is a large gap in knowledge on the early risk mechanisms, but available evidence points to difficulties related to the parents’ sensitivity and response to the infant’s hunger and satiety cues. Particularly, difficulties in breastfeeding and difficulties in the appetite regulation in the parents themselves may lead to early introduction of high energy and fat foods and the parents “overriding” the infant’s internal satiety cues with excess food provision [49–52]. Also, parents’ mental health problems including difficulties of sustained attention, inhibitory control/reward sensitivity, difficulties in delaying gratification, as well as parents’ impulsive eating, may influence their feeding practices [52, 53].

Infants’ regulation of emotions relates to their ability to maintain an affective homeostasis confronted with stressful experiences [25], and problems with emotional regulation are suggested to precede emotional and behavioral problems [17, 29] and emotional eating in childhood [54, 55]. Emotional eating is considered to develop through pathways of physiological stress responses, and through cardiac reactivity influencing appetite [56, 57]. Also, stress exposures may induce hyper- or hypo-activation of the hypothalamic-pituitary-adrenal (HPA) axis, and potentially promote fat accumulation in visceral adipose tissues. These hormonal responses potentially influence appetite and attraction to sweet and fatty foods, mainly by stimulating reward pathways [57, 58]. Research in this field is still scarce, but among the few prospective studies published so far, a study of self-regulation skills and obesity in early childhood has found poor emotional regulation and lower inhibitory control at age 2 years predictive of higher Body Mass Index (BMI) at 5 years of age [59, 60]. This suggests that regulatory problems play a key role in the trajectories of unhealthy weight development.

### Universal and indicated approaches to prevention in infancy

The early onset and the widespread occurrence of both mental health problems and overweight calls for preventive intervention to break the developmental risk trajectories early in the child’s life [61, 62], comprising universal strategies to reduce risk exposures in the population as well as targeted intervention to infants who are most vulnerable [46, 62–67].

General child health surveillance has the potential of universal and indicated prevention of mental health problems as well as unhealthy weight development, conditioned by the availability of feasible and effective methods of intervention [23, 64]. In Denmark, the municipality child health surveillance includes a home-visiting program in which community health nurses (CHN) offer visits to all families on average four times within the first year of the child’s life and thereafter according to the needs of the child and the parents [23, 68, 69]. In this program, which is attended by more than 90% of the population, the CHNs assess child health and development, measure weight and length, and give parents advice regarding e.g. breastfeeding, nutrition, and overall infant care. About 20% of all infant families receive extended services, mostly in the form of elaborated parent counseling, e.g. concerning infant problems of eating and sleeping, excessive crying, the parent-child relation, or parental mental health problems [23]. The CHNs home-visiting program has been a part of the Danish municipality child health for more than 70 years [70], but systematic research in the preventive potentials has been limited so far. Still, an ongoing collaboration between CHNs and researchers has provided CHNs with a standardized record with data stored in a clinical database the *Child Health Database.* This collaboration has initiated a systematic quality improvement of CHNs’ preventive work in the municipalities [71], and sheds light on the needs and challenges for specified prevention regarding mental health [12, 23, 72] as well as unhealthy weight development [47, 48, 55]. Previous research in Danish municipality settings has identified infancy predictors of mental health problems in preschool age that include problems of language, attention, activity, and interests, contact and communication, and problems of eating, sleep, and emotional regulation [12, 23, 41, 55]. These findings have been replicated in studies conducted in other countries and settings [13–17], underscoring the importance of infancy as a key period of preventive intervention to address developmental psychopathology in childhood.

Notably, child age around 9-10 months seems to represent a window of opportunity provided the availability of valid intervention measures [11, 72]. *The PUF-assessment* (PUF in Danish: Psykisk Udvikling og Funktion; in English: Mental Development and Functioning) was developed as a pragmatic service-setting based measure to identify the mental health vulnerabilities seen in young children below two years of age. Specifically, the measure was designed for use at the CHNs’ scheduled home visit at child age about 9 months [73] and manualized to guide the CHNs regarding the child assessments based on parents’ information and clinical observations during the home visit. The evaluations of the CHNs are recorded in the PUF-scheme comprising a total of 28 items that cover motor function, language development, attention, activity, and interests, contact and communication, and regulation of eating, sleep and of emotions. The PUF-assessment has been validated and tested for feasibility [74, 75] and integrated in the PUF-program [76] to guide the CHNs to their action within the existing service settings to address vulnerabilities identified at the PUF-assessment. Face validity and feasibility have been demonstrated, and the program is currently implemented across the municipalities in Denmark.

Still, the PUF-program comprises a baseline approach, and it does not include any standardized intervention to address those infants, who have major cognitive, regulatory, emotional and behavioural problems. These most vulnerable children and their parents are still left behind, without sufficient support to prevent the development of mental health problems.

### Psycho-socially disadvantaged families

Parents’ social, cultural, and psychological resources influence their parenting including how they handle challenges related to the child. A range of parental factors are associated with challenges in providing a healthy and stimulating environment for the child, both regarding the child’s mental health [﻿^7^, ^10^–^12^, ^26^–^30^] and it’s healthy weight development [46, 77–79], particularly, young parental age at the child’s birth, low education, low income, mental health problems, and/or a fragile social network. Unfortunately, the families who face these adversities and are most in need of support, are often difficult to reach in universal efforts [80, 81], and especially so if the CHNs lack knowledge about the intervention to be offered [79, 82].

Anchored in the municipality social services, the CHNs have a primary job of identifying needs of support in accordance with the social legislation in Denmark, and hereby take into consideration the needs of families facing social and economic problems in particular [23]. Still, the challenges experienced by parents with psycho-social disadvantages are often overlooked in the planning of preventive interventions [65, 80, 81]. Limited cognitive, mental, and social resources in parents are thus assumed to influence the fidelity and overall effectiveness of child health public interventions. Ideally, these aspects should be addressed specifically when planning an intervention and be included in the training and supervision of the professionals who deliver the intervention.

### The role of sensitive parenting in the promotion of healthy development in infancy

Parents are the key mediators of healthy development in their child [23, 27, 30, 46, 56, 62, 63] and interventions that improve parents’ sensitivity to the child’s developmental needs are viable means to optimize child mental health [30, 83–85] and probably also a healthy weight development [56, 57].

Programs of home visiting such as the Nurse-Parent-Partnership Program [86] have shown strong potentials of universal prevention of behavioral problems [﻿^30^], particularly among families of psycho-social risk, e.g. among young, single mothers [80, 81, 83, 84, 86]. In Denmark, the home visiting program delivered by CHNs includes approaches in which parents are taught how to meet the infants’ developmentally needs [68]. Several ways of using video feedback to parents are used among Danish CHNs, but no method have been systematically examined regarding the effectiveness to prevent mental health problems in infancy [87, 88].

Regarding feeding, eating and weight, a large part of the CHNs home visits concern health promoting routines regarding feeding and weight [68]. Still, knowledge is limited on effective prevention of overweight in early childhood [62, 89, 90]. The available evidence suggests that improving parents’ sensitivity to the child’s signals and reactions related to hunger and satiety has preventive potential regarding unhealthy weight development, particularly among infants with emotional and behavioral dysregulation related to feeding and eating [56, 91, 92].

Taken together, the promotion of sensitive parenting via specified and structured support from CHNs stands out as a promising avenue to address mental health vulnerabilities in infancy, and, potentially, improve mental health and healthy weight development. Still, the contents of such approaches lack to be systematically explored.

Among methods to promote parenting of vulnerable infants [83, 89–91], the Video-Feedback Intervention to promote Positive Parenting and Sensitive Discipline, VIPP-SD, takes a strong position [30, 92]. Based on attachment theory and theories of social learning [93, 94] it offers a short, focused and highly structured intervention to promote parents’ observational skills and capacity to empathize with the child [95]. The intervention is delivered by health professionals across six to seven highly standardized sessions at home, including education of the parents on topics covering infant and toddler development, using video recordings and feedback to promote parents’ sensitive responsiveness and sensitive discipline [95, 96]. The method has been thoroughly validated [97] and has shown to be modifiable to various areas of infant mental health i.e. regulatory problems [98]; behavioral problems [99], and feeding problems [100]. Also, the VIPP-method has shown to be feasible in various cultural settings and populations, such as families of ethnic minorities, and families of limited cognitive or psycho-social resources [101]. Meta-analyses of Randomized Controlled Trials (RCT) have shown a substantial combined effect size for increased caregiver sensitivity, and a robust combined effect size for improved child outcomes [102], and further, that programs of short duration are more effective compared to longer lasting programs [103].

The VIPP has been developed in the Netherlands, in populations comparable to the Danish, and the fidelity of VIPP-SD has been documented in community settings, also in psycho-socially disadvantage, i.e. families of immigrants and parents with mental health problems or limited cognitive resources [97]. A recent RCT conducted via health visiting services in England found that the VIPP-SD was effective in reducing symptoms of behavior problems in children aged 12 to 36 months [104]. Still, the VIPP-method has not yet been tested on the range of cognitive, emotional, and regulatory vulnerabilities seen in infancy in a pragmatic trial within public health settings.

### Need for the current study

Validated methods to prevent the development of child mental health problems and unhealthy weight within public health settings are much needed, and the potential for a CHNs intervention to promote sensitive parenting of the most vulnerable infants has not been explored yet.

The study will provide urgently needed knowledge on the effect of increased sensitive parenting to infants with developmental vulnerabilities across a range of problems of eating, sleep and emotional regulation as well as problems of development. Furthermore, the study will explore feasible and effective ways to redirect early developmental trajectories of mental health problems and unhealthy weight within public health settings.

### Study Aims

To develop, implement, and evaluate a brief early parenting intervention embedded in the municipality child health care with the overall aim to reduce the risk of enduring mental health problems and unhealthy weight development among vulnerable infants aged 9–24 months.

Specifically, the study aims to.

- create an intervention based on the VIPP-SD method adapted to the PUF-context (the VIPP-PUF intervention) to be implemented from child ages 9 months within the settings of CHNs, and
- examine feasibility, fidelity, and effectiveness of the VIPP-PUF intervention with child mental health and weight at age 24 months as the primary outcomes.

#### Main hypotheses

1. The VIPP-PUF intervention is hypothesized to be feasible for use within the municipality settings of CHNs.
2. Among infants with high levels of cognitive, emotional, and regulatory problems at age 9-10 months, adding the VIPP-PUF intervention to care as usual, will reduce overall mental health difficulties (primary outcome).
3. Among children with high levels of cognitive, emotional, and regulatory vulnerabilities at age 9-10 months, adding the VIPP-PUF intervention to care as usual will promote healthy weight at age 24 months.

#### Secondary hypotheses

1. Among infants with high levels of cognitive, emotional, and regulatory vulnerabilities at age 9-10 months, adding the VIPP-PUF intervention to care as usual will reduce the amount of cognitive, emotional, and behavioral problems at child ages 18 and 24 months.
2. Among infants with high levels of cognitive emotional, and regulatory vulnerabilities at age 9-10 months, adding the VIPP- PUF intervention to care as usual will reduce the development of dysregulation from infancy to age 24 months.
3. Among infants with high levels of cognitive, emotional, and regulatory vulnerabilities at age 9-10 months, adding the VIPP-PUF intervention to care as usual will reduce parents’ experiences of stress and promote cognitions on sensitive parenting and parents’ feeling of competence and relatedness from infancy to age 24 months.

## Methods

### Settings

The study is anchored in the settings of community health nurses (CHNs) in sixteen municipalities across the regions in Denmark. The study municipalities have in total around 7,760 births per year and they are overall representative of the Danish population [105]. Study municipalities are part of the Child Health Database, CHD [106] comprising standardized recordings of information obtained by the CHNs [71], and they have implemented the PUF-program in 2018-2020 [76].

#### Inclusion criteria

To be eligible, the child has been examined at ages 9-10 months in the PUF-program [73–76] and found to have high levels of cognitive, emotional, and regulatory problems, indexed by three or more problems at the PUF-assessment.

#### Exclusion criteria

Children with severe mental or physical disabilities.

Children from families in which neither of the parents speak or understand the Danish or English language.

### Design

**Fig. 1:**
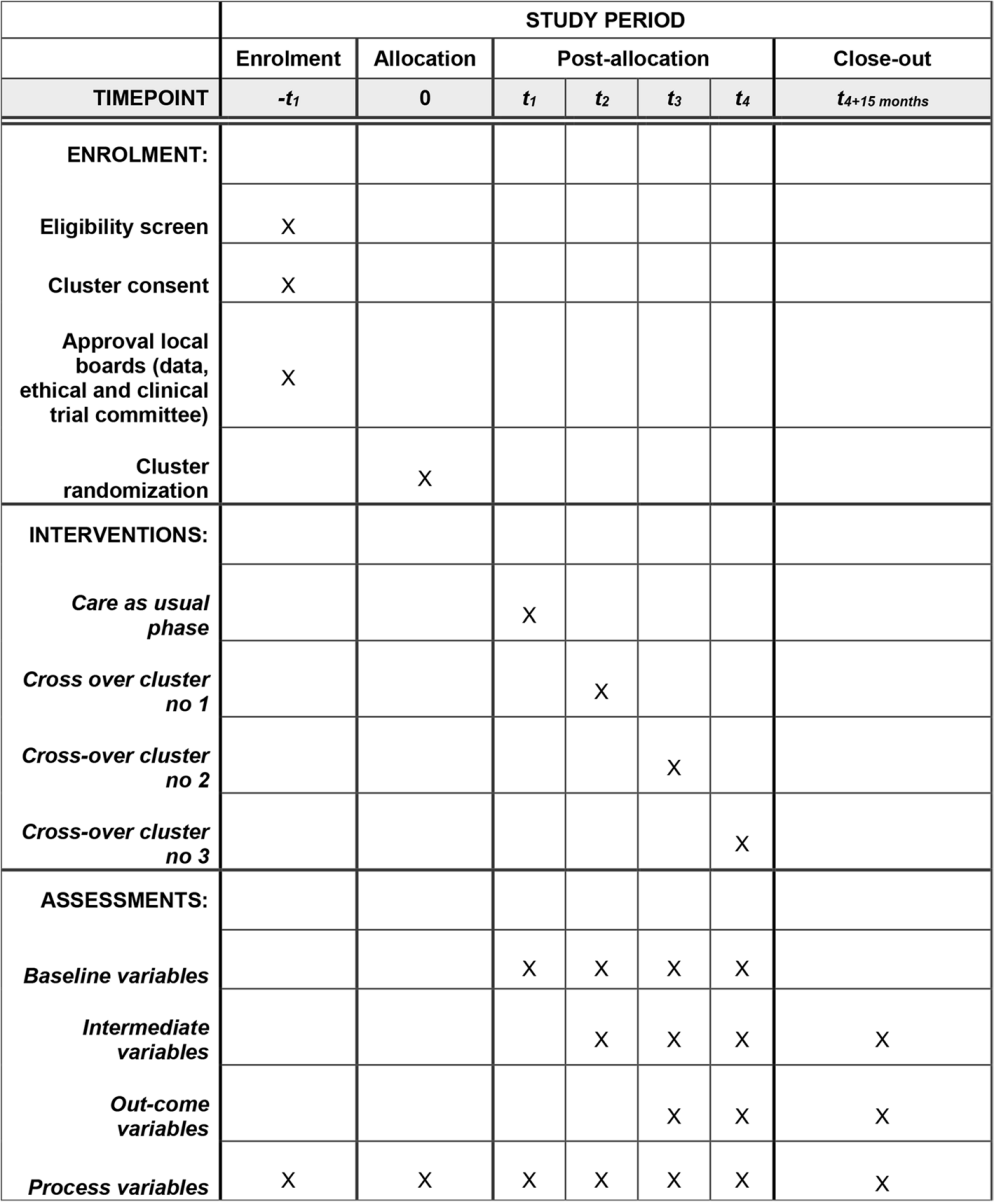
Overview of the Standard Protocol Items: Recommendation for Interventional Trials (SPIRIT) schedule of enrollment, intervention, and assessments

We use a mixed methods approach and the stepped wedge cluster randomized design [107–109] to explore the effectiveness of the VIPP-PUF intervention. We have chosen this design, because it is more feasible to randomize at the municipality level as compared to the level of the individual child or CHN. For practical reasons, all municipalities cannot be trained at the same time but are split into three groups, and with the stepped-wedge design, we randomly allocate the 16 participating municipalities to three clusters defined by the timepoint (t_2_-t_4_) at which their CHNs have been trained and at which they start offering the intervention to families. Also, with the stepped-wedge design, spillover is reduced by the randomization of clusters as compared to individual randomization [107], and interventions are introduced in a stepwise manner to all participating clusters (municipalities in this case), which encourages participation for those who would otherwise have been randomized to the control condition. Further, with the stepped-wedge design control families are recruited in the early phases of the trial (t_1_), which enhances the recruiting of a control group with the level of usual care offered at the beginning of the trial. In the control period participants receive care as usual and sequential random crossover to the intervention cannot be reversed [110]. Figure 1 provides an overview of timepoints for enrollment, intervention, and assessments.

According to the randomization of municipalities, all children assessed to have high levels of cognitive, regulatory, and emotional problems at age 9-10 months (three or more problems at the PUF-assessment) will function as care as usual or current practice controls from t_0_ until the municipalities initiate the intervention in t_2−4_, where new families recruited will function as intervention families.

### Randomization

The 16 municipalities were randomized to initiate intervention at t_1_, t_2_ or t_3_ (Fig. 1). The yearly number of births in each municipality varied greatly, from 118 to 1229 per year (Statistics Denmark 2019). Thus, to ensure an approximately even distribution, randomization was performed in two blocks: The six largest municipalities in one block and the remaining 10 municipalities in another block. Allocation was performed using a blinded custom randomization program by one of the researchers (JT). The result of the randomization program was then handed to an independent investigator not involved in the project who unblinded the randomization.

**Fig. 2:**
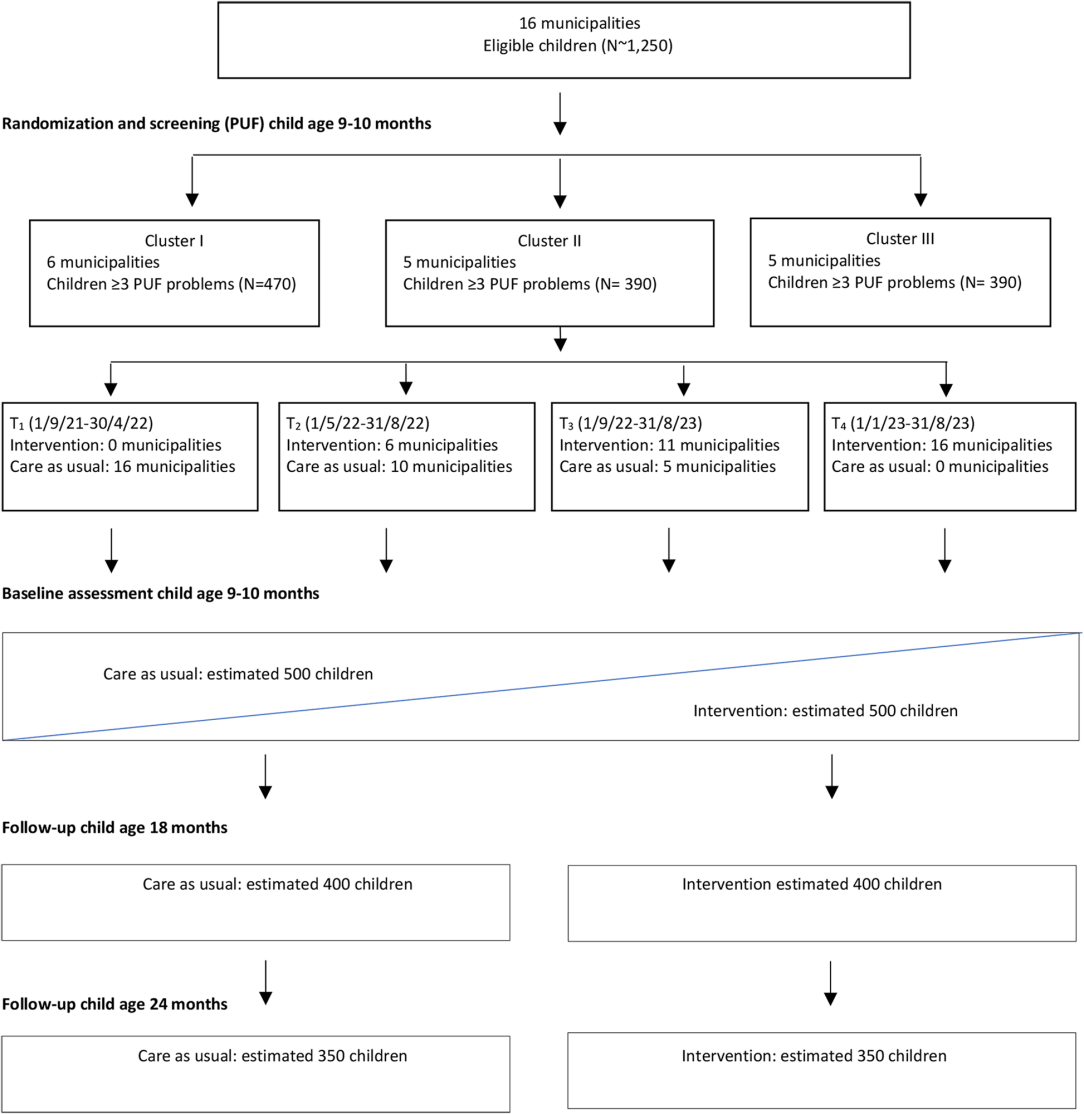
CONSORT diagram of projected participant flow through the Stepped Wedge Randomized Controlled Trial

On a practical level, the 16 participating municipalities were randomly distributed into three clusters (6+5+5), and the clusters were randomized to initiate the VIPP-PUF intervention at three different time points (t_1−4_), according to the stepped-wedge design (Fig. 2).

### Usual care

The CHNs follow the current practice in the municipalities, and specifically at the home visits at child ages 9-10 months, they follow the guidelines of the basic PUF-program and complete the PUF-assessment in dialogue with the parents to offer parents feedback regarding the overall conclusions about the child’s development. According to current practices and routines, the CHNs give parents advices regarding the care of the child, including the needs of referrals to other municipality or primary care settings.

### Recruitment

Eligible children and families are recruited of the CHNs at the home-visit at child age 9-10 months. At this visit, the CHN examines the child using the PUF-assessment and the 28-item PUF-scheme to evaluate the child’s development and function concerning eating, sleep, motor function, language development, attention, contact and communication and emotional regulation [73].

Parents of infants, who show major vulnerability defined as three or more problems at the PUF-assessment are informed about the study by the CHN. The CHN informs about the collaboration between the municipality and the National Institute of Public Health, according to which it is possible to contact families via information from the health services. The CHN leaves written information to the parents for further reading, makes references to the study home page [111], and offers parents assistance to participate if they wish so. Parents are thereafter invited by electronic post (e-Boks) or by postal letter (parents who do not use e-Boks) and further informed about the study and how to participate. They receive a personal log-in to the electronic questionnaires, and they give their informed consent to participate to the project group when completing the questionnaires. The CHNs offer their help to parents who have difficulties in entering their e-Boks or difficulties in completing the questionnaires, at the same home visit or during a visit on another day. Invited parents who have not answered the questionnaires, are send electronic reminders, and after two reminders contacted by the CHN offering help to fill in the questionnaire. Also, in case of incomplete entry of the questionnaires, the parent will be contacted by phone by the project group and offered help to complete the questionnaire. The municipalities are reimbursed from the project for extra time used to inform and assist parents. The parents receive a gift voucher of about 27 euro after answering the questionnaire.

Parents are informed that their participation is fully voluntary, and that they at any time can withdraw from their participation and switch to the usual CHNs’ care in the municipality in accordance with the PUF-program.

### The VIPP-PUF intervention

Parents of infants recruited in the intervention period (intervention cases) are offered the new intervention, the VIPP-PUF. This intervention is created as an add-on to the services offered by CHNs within the current practices.

The development and pilot testing of the VIPP-PUF will be described in a separate paper (Authors, in preparation). In short, the intervention builds on the VIPP-SD program and manual [96], which is adapted to the PUF-context and the municipality settings of CHNs. The VIPP-PUF is co-created in a close collaboration with the developers of VIPP-SD and CHNs from the study municipalities, and takes into account the feedback from parents, who have pilot-tested the VIPP-PUF.

The VIPP-PUF has the following goals:

- To address infants with major vulnerabilities regarding mental health problems indexed as three or more problems of cognitive, emotional, and regulatory functioning at the PUF assessment at age 9-10 months (children with two or less problems receive current practice/ care as usual including the basic PUF-program),
- be acceptable for parents and considering the challenges experienced by families of different kinds of psycho-social disadvantages,
- not induce stress on the participating children and parents,
- be sustainable and easy to integrate with sufficient fidelity into the current practices of the CHNs’ home visiting program.
- adhere to the National guidelines for CHNs in the municipality health care,
- be acceptable and feasible for the CHN, who will be the main responsible for the implementation and delivery of the intervention in the municipalities.

#### The content of VIPP-PUF

Like VIPP-SD, the VIPP-PUF intervention is a highly manualized home-based intervention. The VIPP-PUF manual was created to cover infant development at age 9-14 months and to be delivered over six sessions of about two hours, with two to three weeks intervals by trained and supervised CHNs.

Like VIPP-SD, each VIPP-PUF session involves two parts, the first part involves filming parent-child interactions, e.g., playing together or having a meal together, and the second part involves giving parents focused feedback based on the filmed interactions from the previous session. The first four sessions aim to enhance the parent’s capacity to identify the child’s exploratory behavior, emotional reactions, and attachment cues and to respond to them appropriately, and furthermore to support parents in responding sensitively and consistently to the child’s behavior. In the next two (booster) sessions the key messages are repeated using continuing video interaction material at each session (Authors, in preparation). To ensure the flexibility among the CHNs who deliver the VIPP-PUF intervention, the municipalities will be compensated for the extra time used by the CHNs to complete the intervention, specifically in case of psycho-socially vulnerable and unstructured families.

The VIPP-PUF manual was created January- December 2020 and piloted from January 2021 to July 2021 in four municipalities who are representative for the study municipalities. The pilot study included quantitative as well as qualitative measures (interviews with CHNs and parents), and it suggests overall high fidelity and face-validity.

##### Education of community health nurses﻿

The VIPP-PUF intervention includes a program of training and supervision, which has been developed from the VIPP-SD program [95, 96] in close collaboration with the developers of VIPP-SD and certified trainers from the VIPP-SD Institute in Leiden, the Netherlands. The program of training and supervision has been piloted in four study municipalities and among eight CHNs. In the study period, about 50 CHNs will be educated with a minimum two CHNs per municipality.

#### Development of the VIPP-PUF intervention components

The specific tools and activities of the intervention has been created in dialogue with the Advisory group of leading CHNs and the trainers from the VIPP-Institute and adjusted in accordance with the findings from the pilot study, which includes feedback from CHNs as well as parents. Educational experts, graphic- and web-designers, videographers and writers are currently involved in the study home page [111], extended scripts, plans, visual and written outlines (booklets, leaflets/handouts/newsletters).

### Measures

Assessments are conducted at baseline (child ages 9-10 months) and at child ages 18 and 24 months. See Table 1 for an overview of measures included at baseline, intermediate and follow-up assessments.

**Table 1:** Measurement at baseline assessment (child ages 9-10 months), post-test assessment (child ages 18-19 months) and follow-up assessment (child ages 24-25 months)

Measures (Child)	9-10 months	18-19 months	24-25 months
PUF-assessment﻿	x		
Ages and Stages Questionnaire, Social Emotional 2; ASQ:SE2	x	x	x
Child Behavior Checklist; CBCL		x	
Strengths and Difficulties Questionnaire; SDQ			x
BMI z-scores	x		x
Feeding and Eating behavior Questionnaires		x	x
Video-recordings of mealtimes		x	x
Video-recordings of parent-infant interaction		x	x
Child Interaction Behavior; CIB		x	x
**Measures (Parents and parent-child relations)**			
Mother and Baby Interaction Scale; MABISC	x		
Being a Mother; BaM13		x	x
Parental Stress Scale; PSS	x	x	x
WHO-5 well-being index; WHO-5	x	x	x

*The Ages and Stages Questionnaire, Social-Emotional 2, ASQ:SE 2*, version for children aged 1 to 60 months [112]. *The ASQ:SE 2* [112] completed by parents is used to measure child self-regulation, compliance, communication and adaptive functioning, and social and emotional function at age 18 and 24 months. ASQ-SE comprises 19 to 33 items rated by parents and includes a box in which parents check if the behavior is a concern for them. The ASQ:SE2 is well-validated and the most frequently used measure of young children’s social and emotional development, internationally as well as in Denmark [113].

The *Child Behavior Checklist, CBCL-*version for children aged 1 ½ -5 years completed by parents [114] is used to measure problems of eating, sleep, emotional problems, behavioral problems, and problems of hyperactivity, concentration, communication, language, and social interaction at 18 months. The *CBCL 1 ½ -5 years* comprises 99 items, it is a quite long questionnaire that might be challenging for parents with limited cognitive resources to complete. However, the CBCL 1 ½ -5 years is the most comprehensive measure to the overall screening of mental health problems in children as young as 18 months, and for the Infant Health study particularly relevant because the inclusion of markers of cognitive problems, as well as overeating and measures of dysregulation [114]. Notably, CBCL 1 ½ -5 years has been found predictive of persistent emotional and behavioral dysregulation in preschool to school age [﻿^115^]. It has been used in several population-based studies in Denmark [6, 11, 23, 74], and also in studies in which CHNs interview parents [11, 23, 74]. Building on experiences from previous research, we include CHNs assistance to help parents who have difficulties in completing the CBCL.

*The Strengths and Difficulties Questionnaire, SDQ*, the version to be completed by parents [116] (www.sdqinfo). The SDQ parent version is a brief and widely used measure assessing emotional difficulties, conduct problems, hyperactivity, peer relationship problems, and prosocial behavior. It includes 25 items which require the respondent to rate the statement regarding the child over the last 6 months (Not true, some-what or sometimes true, very true or often true). SDQ total scores have been found predictive of persistent child mental health problems [115]. The SDQ has been validated for use in children down to the age of 2 years [117, 118]. Moreover, the SDQ has been found suitable for the prospective investigation of mental health problems from 24 months and onwards; it is used in epidemiological research worldwide [119], and also in Danish populations, with Danish norms being available [120] (www.sdq.dk).

*BMI z-scores* [121] based on measurements of weight and lengths at home visits at child ages 24 months using transportable scales and height carts and guidelines on how to perform the measurements. Supplementary measures on healthy weight development will be added.

*Video-recordings of mealtimes* at child ages 24 months will be used to examine the child’s eating behavior, the parents feeding behavior, and the parent-child interaction regarding parental sensitivity, intrusiveness and limit setting, regarding the child’s involvement, withdrawal and compliance and concerning the overall dyadic reciprocity during a meal. The final measures of recordings and coding are currently pilot tested.

*Parent questionnaire on eating behavior* at 18 and 24 months will include questions on parental feeding as well as child eating behavior, including emotional eating, impulsive eating, appetite regulation and overeating. The final measures are currently pilot tested.

*Video-recordings of parent-infant interaction at age 18 months and 24 months* will be based on a subsample to examine the parent-child relationship and in particular, sensitive parenting using observer-ratings based on the Child Interaction Behavior (CIB) system (139; 140). The CIB system contains 22 parent behavior codes, 16 child behavior codes, and five dyadic codes which can be aggregated into the following composites: sensitivity, intrusiveness, limit setting, involvement, withdrawal, compliance, dyadic reciprocity, and dyadic negative states. The CIB system has been validated in normative as well as high-risk populations, and shows stability over time, predictive validity, and adequate psychometric properties [139, 141–143]. We will include the CIB- coding in the coding of mealtime observations. Coders will be trained to intercoder reliability ICC > 0.65, Pearson’s r > .70, and regular meetings and checks will be organized to prevent coder drift.

*The Mother and Baby Interaction Scale, MABISC* [122] is an 10 items measure for the early screening of the quality of the parent-infant relationship (child age <13 months). It includes items reflecting the parent’s reactions to their infants in different daily situations covering the last two to three weeks, in which they can agree or disagree. The scale is rated from 0 to 4 on a 5-point rating scale (always, most of the time, occasionally, not often, never); and values are summarized to a total score with low total scores indexing better interaction quality.

*The Being a Mother (BAM-13)* [123] is a 13-item measure of parent’s satisfaction and experiences of being a parent. Items include statements on daily situations related to parenthood within the last two to three weeks; they are rated on a 4-point rating scale (no, hardly ever; no, not very often; yes, some of the time; yes, most of the time). The total score range is 0-39, with a low score indicating higher satisfaction.

T*he Parental Stress Scale, PSS* [124] is an 18-item measure of parenting stress associated with raising children. Respondents rate their perception of stress related to both positive and negative statements. The statements are rated on a 5-point rating scale (strongly disagree, disagree, undecided, agree, strongly agree). Total scores range between 18 and 90, where a low score indicates less stress. The PSS has been validated and found reliable for use among parents of differing background [124], and recently also among Danish parents of children aged 0-1 year [125] and 2-18 years [126].

T*he WHO-5 well-being index, WHO-5* [127] is used to explore parental factors. WHO-5 is a short questionnaire consisting of 5 questions measuring subjective psychological well-being. The statements are rated on a 5-point rating scale (aAll of the time; most of the time; more than half the time; less than half the time; some of the time; at no time). The scores range between 0 and 25 and are multiplied by 4 to give the final score from 0 representing the worst imaginable well-being to 100 representing the best imaginable well-being [128].

#### Data on the child’s physical health and the social-economic conditions of the family

Data on pregnancy, birth, perinatal factors, and physical and mental disorders diagnosed at hospital are obtained from the Medical Birth Registry and Danish patient registries [129].

Information on ethnicity, parental age, family composition, parents’ education, and family economic position are obtained from Danish population registries [129, 130]. Information on CHNs assessments of weight and length, psycho-motor development, regulatory problems (eating, sleep, excessive crying), parent-child relation problems, and parental mental health problems recorded by CHNs at home visits between the child’s birth and age 9 months are obtained from the Child Health Database [71, 72].

#### Data management and data protection

Quantitative data is collected following a standard data management protocol for data entry and through a secured online survey database (RED Cap –Research Electronic Data Capture) hosted at OPEN Storage, OPEN, Open Patient data Explorative Network, Odense University Hospital, Region of Southern Denmark [131]. Participants receive a letter with a direct link to the questionnaire in e-Boks, a digital mailbox system providing all Danish citizens with a private e-mail account tied to their social security number. Danish public agencies use e-Boks as a secure platform for digital communication with citizens (see www.e-boks.com). A trial monitor will continuously check data quality. The study is registered and approved by the University of Southern Denmark in accordance with the Data Protection Regulation and the General Data Protection Regulation (GDPR) (EU) 2016/679; Notification number: 11.090. Access to data will be restricted to the research team.

#### Statistical analyses

The effectiveness of the VIPP-PUF intervention is measured as differences in SDQ total difficulties scores between infants receiving the VIPP-PUF intervention compared to infants receiving care as usual.

Comparisons between the children and parents receiving care as usual, and children and parents receiving the VIPP-PUF intervention will use the intention to treat principle, where all the children are analyzed according to their allocation status. Random effects linear regression models will be fitted to compare means for continuous outcomes (including the primary outcome measured as the SDQ total difficulties score) and random logistic regression will be fitted to compare binary outcomes (e.g. border line/abnormal versus normal SDQ total score) between care as usual and children receiving VIPP-PUF-intervention, allowing for the correlation between outcomes of children from the same municipality. Prognostic factors at baseline will be included: child factors: sex, birth parameters, BMI z-scores from birth to age 9 months, regulatory problems from ages 2-9 months, and ASQ:SE2 score at 9-10 months; family factors including parents’ age, educational level, ethnicity, and parental mental health problems; and at the municipality level, e.g., level of social disadvantage.

Interaction terms will be included to explore possible differences in intervention effect (on the primary outcome SDQ total difficulty score) between gender, pre-defined subgroups based on BMI z-scores 0-9 months, low versus high scores of regulatory problems 2-9 months (0-2 problems versus ≥3 problems of eating, sleep, or emotional dysregulation); low versus high baseline ASQ:SE2 scores and parental characteristics. Also, additional analyses of effect modification will further explore the effect by different population groups (socio-economic position, family structure and migration status).

**Fig. 3:**
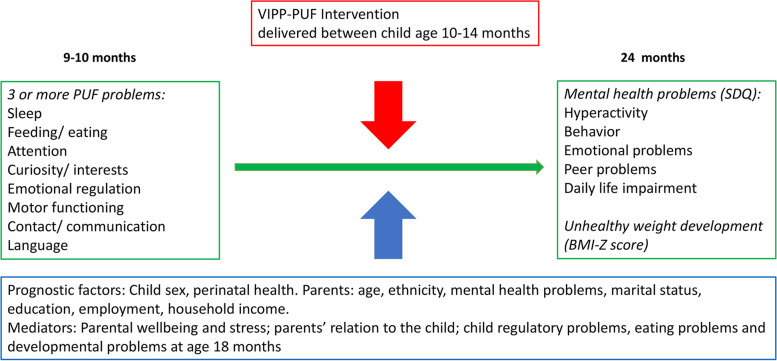
Diagram of hypothesized paths

We will use path analysis to explore whether the effect of the intervention on BMI z-score at age 18 and 24 months are mediated through regulatory problems and parental characteristics. Using path analysis, we can assess the direct effect of the intervention, corresponding to the proportion not mediated through risk factors for child overweight, and the indirect effects of the intervention, corresponding to the proportion mediated through selected risk factors for childhood overweight. The total effect is the sum of the direct and indirect effects. For an overview of the hypothesized paths see Fig. 3.

Specifically, we will explore weight trajectories from birth to age 24 months to estimate the effects of the VIPP-PUF intervention on weight development. We will adjust for BMI z-score at birth and examine the role of pre- and postnatal risk factors (including gestational age, birth weight, congenital diseases) and problems of feeding, eating, sleep and emotional reactivity between ages 2 and 6 months.

#### Sample size considerations

Based on a sample size of about 1,000 participants, we have estimated that a potential loss to follow-up may be in the order of 20-30%, which leaves 700-800 parent-child dyads available for full follow-up at 24 months. We expect that about 350-400 of them have received the VIPP-PUF intervention and 350-400 care as usual. We expect that the mean SDQ total difficulties score in children with ≥3 problems is about 13 [120] and that it will be reduced by the intervention to a total difficulties score of about 11 [119]. Further, we expect that the standard deviation of the SDQ total difficulties score is 8 (www.sdqinfo.org). Uncorrected for clustering and repeated measurements, the sample size will allow 80% power to detect a standardized effect at the significance level of 0.05. The intra-cluster correlation coefficient (ICC) for the SDQ total difficulties score is anticipated to be no higher than 0.05.

Taking the design effect of the stepped wedge design into account, a sample size of 690 is required to detect the anticipated effect, as the stepped wedge design could reduce the required sample size in cluster randomized trials [108]. As we expect that it is possible to enroll and obtain full follow-up on at least 700 children, the power in the study allows for testing moderator effects.

### Process evaluation

The process evaluation is nested within the Infant Health Study following the principles of intervention Mapping [132]. Mixed methods will be used to explore and analyze the implementation process to obtain knowledge about activities and effects, and to extend the understanding of the relationship between the intervention and the outcome [133, 134].

We will evaluate whether the intervention is implemented as intended (fidelity), and whether and to what degree it has reached all families within the intended target group and across various risk groups (reach), with a specific focus on families of psycho-social disadvantage. Also, we will assess the acceptability of the intervention among CHNs and parents, and their appreciation of and satisfaction with the intervention program, to get a deeper understanding of how, why and for whom the intervention is effective or not [135].

The process evaluation runs in parallel to the effect study, and includes questionnaires and interviews of the CHNs, parents and stakeholders to explore the fidelity and reach of the intervention.

The evaluation of the process of creating and pilot-testing the VIPP-PUF intervention will be described in a separate paper.

### Components of the process evaluation

The VIPP-PUF is a pragmatic and complex service setting based intervention, and the process evaluation will include mixed methods to evaluate the multiple aspects of the implementation of the intervention [134], such as (1) the context (including documentation of recruitment efforts that may influence the intervention implementation); (2) the reach (including whether the VIPP-PUF intervention has reached all families across various risk groups); (3) the dose delivered (including the amount of intended units of the intervention delivered or provided); (4) the dose received (including the extent to which participants engage with and receive the intervention and materials); (5) fidelity (including the extent to which the intervention was delivered as planned, representing the quality and integrity of the intervention conceived by the developers) and (6) implementation. Also, we will explore procedural aspects, such as the variability allowed in the different municipalities, and among CHNs, as based on the child and the family [133, 134, 136].

Importantly, the COVID -19 pandemic has been ongoing since March 2020, and the process evaluation will include the aspect of this natural experiment on the overall implementation.

*Quantitative data* relating to the administration and delivery of the CHNs’ educational and training courses include recordings of the adaptation of program skills and self-monitoring checklists for each intervention session. In order to reflect the overall implementation and impact of implementation on mental health problems and weight, all main components of the VIPP-PUF intervention will be addressed according to the guidelines elaborated in the pilot-study, e.g. specified videotape during daily situations at home-visits; themes of the program delivered for each session and successively elaborated during the intervention period according to the guidelines; the CHNs’ use of the manualized instruction for feedback to the parent according to each theme of the program; and delivery of written material on sensitive parenting and sensitive responding in daily situations about e.g. eating and feeding [137]. Questionnaire surveys will be set up to collect information on contextual factors including competing health promotion activities targeting families with infants.

*Qualitative data* will be collected to inform the intervention processes, to explore the translation of the CHNs’ intervention skills into practice and CHNs experiences of using the intervention, and to investigate how parents experience and respond to the intervention. Data will be collected using focus groups [﻿^138^] with CHNs, and individual semi-structured interviews [139] with participating parents. We will conduct participant observations [139] at a selected number of home visits at different times throughout the study, to obtain knowledge about the actual use of VIPP-PUF in the home visit, and how parents respond. Semi-structured interviews with mothers and fathers will be used to explore their experience of the intervention, e.g., their acceptability of the intervention, and their hopes and priorities for change in relation to the intervention provided. At the municipal level, individual and focus group interviews [﻿^138^] will be conducted to further investigate barriers at the organizational level.

The qualitative part of the evaluation includes telephone interviews with National and municipality health and organizational stakeholders e.g., heads of health departments, health administrators, health planners, and managing CHNs, to collect data on differential use of CHNs’ services, the attribution to the CHNs being trained, and the perception of the settings of the intervention among other available sources of support.

*Interview guides* will be developed for the focus groups and the semi-structured individual interviews. Interviews will be audio-recorded and transcribed verbatim and anonymized [140].

A structured observation guide will be developed for the participants observations, and detailed field notes will be produced based on this guide.

#### Analyses of qualitative data

The empirical material (i.e. transcriptions of interviews and field notes) will be analyzed following general principles for qualitative data analysis [141, 142﻿]. A systematic coding approach will be used to identify important themes across the empirical material to be coded and grouped into categories of overall themes. Recurrent topics will be identified, compared, and categorized. The analytical process will be inspired from collaborative data analysis [143], through which different perspectives are brought to bear on the analysis and interpretation of the data. The analysis and interpretation of results will be brought into dialogue with existing research to strengthen validity and generalizability.

Thematic analyses of interview and focus group data will be driven by the research question, but it will allow for more inductive analysis whereby novel and emergent themes are also identified [141].

Summary and illustrative data relevant to the aims of the quantitative and qualitative research for each phase in the different parts of the study will be available, to facilitate further interpretation and discussion of which processes worked well or not so well within the study.

### Health economic evaluation

The planned health economic evaluation will take a broad public sector perspective, including the use of all health, education, and social care services. Data will be presented by sector to allow alternative perspectives to be considered separately. The evaluation of the cost-effectiveness of the VIPP-PUF intervention will include data from Danish national registers and data on service use from the Child Health Database.

### Timeline

The project was planned to be conducted from January 2020 through December 2024. However, the outbreak of the COVID-19 pandemic has had large impact on the CHNs’ daily work forcing, leaders and practicing health nurses to functions associated with the control of the epidemic in Denmark. Accordingly, the initial phases of the study have been delayed, including the development and pilot-testing of the VIPP-PUF; the primary education and training of the CHNs; and the establishment and pilot-test of recruitment procedures, the research database and baseline questionnaires, all firstly completed summer 2021 with a delay of in total 6 months. Correspondingly, the recruitment phase will be postponed with estimated 6 months.

Overall, the timeline of the study is estimated to be extended with a total of 12 months, to December 2025.

### Dissemination

Stakeholders, including CHNs delivering the intervention, and other collaborators, will receive regular updates on the trial. Important protocol modifications (e.g., changes to eligibility criteria, outcomes, analyses) are communicated to participating municipalities, CHNs, www.ClinicalTrials.gov and at the study home page www.smaa.boerns.sundhed.dk.

The results of the trial will be disseminated to participants, healthcare professionals, researchers and to the public. Study reports will be submitted to the trial funders at regular intervals to monitor progress, including a final report at the end of the trial. The trial team will present the progress and results of the study at relevant national and international conferences to both research and clinical audiences. Also, the results will be disseminated to the public via the home pages and public medias.

We have pre-registered the study protocol, and expect to publish 10-12 scientific manuscripts, including the study design paper, a paper on the development and pilot-testing of the VIPP-PUF intervention, papers on process evaluation psycho-social disadvantaged families (three papers), and papers on effect evaluation within the areas explored, mental health (three papers), weight (three papers) to be submitted to international peer-reviewed journals.

All publications follow the Vancouver declarations on authorship and comply with the SDU Open Science Policy including Open Access publishing of results.

### Study management

The Infant Health study is hosted at the National Institute of Public Health, NIPH, University of Southern Denmark, SDU and conducted in close collaboration with the participating municipalities, including an *Advisory group of leading CHNs* from the sixteen participating municipalities with additional informal contact between meetings.

The *Trial management group* including members of the project group, the direction of the National Institute of Public Health, NIPH and leading health nurses meet from two to four times a year to monitor and support the progress of the study.

#### Trial and data steering committee (TDSC)

The Trial and Data Steering Committee (TDSC) includes four external members independent from investigators and the sponsors. The external members are affiliated respectively, the Department of Public Health, Section for Health Promotion and Health Services Research, University of Aarhus, Department of Public Health, Section of Epidemiology, University of Copenhagen, and Institute of Clinical Medicine, Faculty of Health Sciences, University of Copenhagen. The TDSC provides the overall supervision of the trial and assess the progress of the trial, the safety of data, and the critical effectiveness endpoints. Specifically, the TDSC monitors the progress of the trial and advises on scientific credibility, monitor the accumulating trial data, and make recommendations as to whether the trial can continue, or underline if there are any ethical or safety issues that may necessitate a modification to the protocol or closure of the trial, or if there is evidence of systematic recruitment or attrition bias. The TDSC ultimately carries the responsibility for deciding whether the trial need to be stopped on grounds of effectiveness or safety. The TDSC meets two to three times annually.

### Study status

Randomization into Cluster I, II and III was completed February 2021. Recruitment and baseline assessments of current practice families in all clusters commenced August 2021. Intervention was planned to commence January 1, 2022, but is postponed to May 1, 2022, Cluster I; September 1, 2022, Cluster II; and January 1, 2023 Cluster III. The 18 months follow-up commences in May 2022 and the 24 months follow-up assessments will begin in November 2022. Recruitment is due to be completed in April 2023. At the time of publication, the trial was being implemented as per Protocol Version 1.0, dated October 2020.

Any important protocol modifications will be communicated and recorded at the study home page www.smaa.boerns.sundhed.dk and www.ClinicalTrials.gov.

## Discussion

This project is the first to examine mental health and weight outcome at 24 months of a pragmatic service-setting based intervention addressing sensitive parenting of infants with cognitive, emotional, and regulatory vulnerabilities at child ages 9-10 months.

The project is innovative in the systematic approach to the prevention of frequent and potentially impairing health problems in infants from the general population. Using the mixed methods process evaluation including comprehensive quantitative and qualitative data and a randomized controlled trial design, the project will provide important knowledge on future effective, individually targeted and much needed public health interventions to address mental health problems and unhealth weight development in early childhood.

### Research perspectives - The Infant Health Birth Cohort

In parallel with the Infant Health Study focusing on the most vulnerable children, a birth cohort comprising all children born in the study municipalities in the study recruitment period is planned to be investigated.

Based on the estimates on births per year in the study municipalities and a recruitment period of 24 months, a total of about 15,682 infants will reach 9-10 months in the study period, of whom about 12,550 (80%) are estimated to be assessed using the PUF-program at CHNs’ home visits at age 9-10 months. Of these, estimated about 1,255 (10%) will show three or more major cognitive, emotional, or regulatory problems. Assuming a recruitment rate of 80% as estimated from previous work, a sub-cohort of about 1000 at-risk children will be nested in the entire cohort of about 12,550 children. Both cohorts are planned to be followed longitudinally, using comprehensive data from the municipality Child Health Database and the Danish population registries, thus extending the results from the primary Infant Health study.

## Supplementary Information

**Additional file 1.**

**Additional file 2.**

**Additional file 3.**

**Additional file 4.**

**Additional file 5.**

**Additional file 6.**

## Data Availability

The datasets generated and analyzed during the current study will not be publicly available because they contain sensitive material, identifying participant information. Permission to access data for scientific purposes on reasonable request is possible for researchers meeting the criteria for access to confidential information. For researchers in a European country, permission to access these data for scientific purposes can be obtained from the personal data agency SDU Research & Innovation (RIO), University of Southern Denmark. Information on how to apply for data access can be obtained by emailing kd.uds@atadnosrep.uds. For researchers outside a European country, permission to data access can be obtained for scientific purposes on reasonable request from the Danish Data Protection Agency by emailing kd.tenyslitatad@td.

## Abbreviations

ADHD: Attention Deficit Hyperactivity Disorder
ASQ: SE2:Ages and Stages Questionnaire – Social Emotional 2
BaM-13: Being a Mother-Questionnaire 1
BMI: Body Mass Index
CBCL 1½-5: Child Behavior Check List 1½-5 years
CHD: Child Health Database
CHN: Community Health Nurse
CIB: Child Interaction Behavior
CONSORT: Consolidated Standards of Reporting Trials
COVID-19: Coronavirus disease 2019
MABISC: Mother and Baby Interaction Scale
PUF: Mental Development and Function (In Danish:Psykisk Udvikling og Funktion)
PSS: Parental Stress Scale
RCT: Randomized controlled trial
SDQ: Strengths and Difficulties Questionnaire
SPIRIT: Standard Protocol Items:Recommendations for Interventional Trials
TDSC: Trial and Data Steering Committee
VIPP: Video-Feedback Intervention to Promote Positive Parenting
VIPP-PUF: Video-Feedback Intervention to Promote Positive Parenting and Psykisk Udvikling og Funktion (In English:Mental Development and Function)
VIPP-SD: Video-Feedback Intervention to Promote Positive Parenting and Sensitive Discipline
WHO-5: World Health Organisation-5 well-being index

## Declarations﻿

The CHNs inform parents about the collaboration between the municipality and the National Institute of Public Health, according to which it is possible to contact families via information from the health services. Parents are informed by written information attached the electronic invitation (e-Boks) or a postal letter (to parents who do not use e-Boks) with a personal log-in to the electronic questionnaires, and they give their informed consent to participate to the project group via the survey database, when they have completed the electronic questionnaires. They parents are informed that their participation is fully voluntary, and that families who do not wish to participate, receive current practice or care as usual. It is underscored that at any stage of the project, the parents can withdraw their participation, and then receive current practice. Notably, the study concerns a specified intervention as an add-on to existing services provided by CHNs in the study municipalities [144]. If the CHNs observe potentially adverse effects of the VIPP-PUF intervention, this will be recorded to the study team, and in accordance with the current practices and the PUF-program, the CHNs switch on the care as usual. Importantly, in the existing routines and the current practice of CHNs, about 20% of families receive extended childcare in accordance with the social legislation. More than 90% of the families use the services offered by the CHNs, which include counselling and information on sensitive issues related to challenges of the health and well-being of the child, e.g., mental health and overweight. In this study, the CHNs use a more standardized and specified method to address the needs of the most vulnerable infants and their families, of which the majority already have close contact with the CHNs as a part of the existing routines. Also, video feedback, which is a core ingredient of the VIPP-PUF intervention, is already frequently used among Danish CHNs as a way of communicating infants’ needs to the parents, and previous experiences show that the video feedback approach is well accepted among parents and regarded as a helpful supplement for the parents to understand the CHNs’ guidance (87).
