# Publisher Correction: The Infant Health Study - Promoting mental health and healthy weight through sensitive parenting to infants with cognitive, emotional, and regulatory vulnerabilities: protocol for a stepped-wedge cluster-randomized trial and a process evaluation within municipality settings

**DOI:** 10.1186/s12889-022-12677-0

**Published:** 2022-02-21

**Authors:** Anne Mette Skovgaard, Marian Bakermans-Kranenburg, Maiken Pontoppidan, Tine Tjørnhøj-Thomsen, Katrine Rich Madsen, Ida Voss, Stine Kjær Wehner, Trine Pagh Pedersen, Lotte Finseth, Rodney S. Taylor, Janne Schurmann Tolstrup, Janni Ammitzbøll

**Affiliations:** 1 National Institute of Public Health, NIPH, University of Southern Denmark, Copenhagen, Denmark; 2 Clinical Child and Family Studie, Vrije Universiteit, Amsterdam, The Netherlands; 3 The Danish Center for Social Science Research, VIVE, Copenhagen, Denmark; 4 MRC/CSO Social and Public Health Sciences Unit & Robertson Centre for Biostatistics, Institute of Health and Well Being, University of Glasgow, Glasgow, UK; 5 University of Exeter, Exeter, UK

**Correction to: BMC Public Health 22,1–19 (2022)**

**https://doi.org/10.1186/s12889-022-12551-z**

During the publication of the original article the symbols in Table 1 were incorrectly placed as a result of a typesetting error. The incorrect (Table 1) and correct (Table 2) table are available in this correction article. The publisher apologizes to the authors and readers for the inconvenience.

**Table 1:** Incorrect version of table 1 as originally published

Measures (Child)	9-10 months	18-19 months	24-25 months
PUF-assessment	x	x	x
Ages and Stages Questionnaire, Social Emotional 2; ASQ:SE2	x		
Child Behavior Checklist; CBCL		x	
Strengths and Difficulties Questionnaire; SDQ			x
BMI z-scores	x		x
Feeding and Eating behavior Questionnaires		x	x
Video-recordings of mealtimes		x	x
Video-recordings of parent-infant interaction		x	x
Child Interaction Behavior; CIB		x	x
**Measures (Parents and parent-child relations)**			
Mother and Baby Interaction Scale; MABISC	x		
Being a Mother; BaM13		x	x
Parental Stress Scale; PSS	x	x	x
WHO-5 well-being index; WHO-5	x	x	x

**Table 2:** Correct version of table 1

Measures (Child)	9-10 months	18-19 months	24-25 months
PUF-assessment	x		
Ages and Stages Questionnaire, Social Emotional 2; ASQ:SE2	x	x	x
Child Behavior Checklist; CBCL		x	
Strengths and Difficulties Questionnaire; SDQ			x
BMI z-scores	x		x
Feeding and Eating behavior Questionnaires		x	x
Video-recordings of mealtimes		x	x
Video-recordings of parent-infant interaction		x	x
Child Interaction Behavior; CIB		x	x
**Measures (Parents and parent-child relations)**			
Mother and Baby Interaction Scale; MABISC	x		
Being a Mother; BaM13		x	x
Parental Stress Scale; PSS	x	x	x
WHO-5 well-being index; WHO-5	x	x	x

