# Supplementary material for: The Infant Health Study - Promoting mental health and healthy weight through sensitive parenting to infants with cognitive, emotional, and regulatory vulnerabilities: protocol for a stepped-wedge cluster-randomized trial and a process evaluation within municipality settings
Source: BMC Public Health. 2022 Jan 28;22:194. doi: 10.1186/s12889-022-12551-z (PMC8796192; doi:10.1186/s12889-022-12551-z)
Supplement: Supplementary file 2 — Additional file 2. [file 12889_2022_12551_MOESM2_ESM.docx]

**A2. Overview of trial registration data**

| Data category | Information |
| --- | --- |
| Primary registry and trial identifying number | [www.ClinicalTrials.gov](http://www.ClinicalTrials.gov); ID NCT04601779; Protocol ID 95-110-21307 |
| Date of registration in primary registry | 25 June 2021 |
| Secondary identifying numbers | - |
| Source of monetary or material support | National Institute of Public Health, University of Southern Denmark. |
| Primary sponsor | Novo Nordisk Foundation |
| Secondary sponsor | Independent Research Fund Denmark |
| Contact of public queries | <https://www.sdu.dk/en/sif/forskning/projekter/smaa_boerns_sundhed>  smaaboern@sdu.dk |
| Contact for scientific queries | <https://www.sdu.dk/en/sif/forskning/projekter/smaa_boerns_sundhed>  smaaboern@sdu.dk |
| Public title | Infant Health |
| Scientific title | Infant Health – Supporting infants’ mental health and healthy weight development through community health nurses’ promoting sensitive parenting. |
| Countries of recruitment | Denmark |
| Health conditions of problems studied | Mental health problems and unhealthy weight in early childhood |
| Intervention | A complex service setting-based intervention including the VIPP-PUF (Video-feedback to promote Positive Parenting - PUF) |
| Key inclusion and exclusion criteria | 1. Children with severe mental or physical disabilities  2. Children whose parents have major problems in speaking or understanding Danish or English |
| Study type | A stepped-wedge cluster-randomized trial and a mixed method process evaluation |
| Date of first enrolment | 16 August 2021 |
| Target sample size | 900-1000 children (450-500 care as usual controls and 450-500 intervention cases) |
| Recruitment status | December 2021: still recruiting controls |
| Primary outcome | Reduction in mental health problems (SDQ total difficulties score) at child age 24 months. |
| Key secondary outcomes | 1. Promotion of healthy weight at age 24 months 2. Reduction of infant cognitive, emotional, and behavioural problems at child age 18 and 24 months 3. Reduction of development of dysregulation from infancy to 24 months 4. Reduction of parents’ experiences of stress at child age 24 months 5. Promotion of parents’ feeling of competence and relatedness from infancy to age 24 months. |
