# Supplementary material for: The Infant Health Study - Promoting mental health and healthy weight through sensitive parenting to infants with cognitive, emotional, and regulatory vulnerabilities: protocol for a stepped-wedge cluster-randomized trial and a process evaluation within municipality settings
Source: BMC Public Health. 2022 Jan 28;22:194. doi: 10.1186/s12889-022-12551-z (PMC8796192; doi:10.1186/s12889-022-12551-z)
Supplement: Supplementary file 3 — Additional file 3. [file 12889_2022_12551_MOESM3_ESM.pdf]

18-06-2020
Notification number: 11.090

Opinion - Små børns sundhed / Infant health

Dear Anne Mette Skovgaard,

On 07-05-2020 you have submitted a research project to SDU RIO.

The notification shows the project to be about:

Formålet med dette projekt er at udvikle og afprøve en intervention, som 1) adresserer mental sårbarhed hos det enkelte barn, 2) er baseret på at opruste forældrenes forståelse og håndtering af barnets sårbarhed, 3) som er baseret på videnskabelig evidens, og 4) som integrerer danske sundhedsplejerskers erfaringer, og 5) er realistisk gennemførlig indenfor den eksisterende sundhedspleje i kommunerne.

Projektet handler om forebyggelse af mentale helbredsproblemer hos småbørn, og omfatter udvikling og pilotafprøvning af en specifik indsats til de mest sårbare småbørn samt et randomiseret kontrolleret studie af interventionens effekt.
.

The project is expected to be completed on 30-06-2025 and the personal data will be deleted, anonymised or archived no later than31-01-2030. Please note that the personal data must not be stored for longer than necessary for the purposes for which the information was collected.

As you have stated that personal data is being processed within the project, this e-mail will also be sent to IT-Service employee, Erik B. Madsen, who will help find an IT solution that ensures the appropriate level of security.

Your notification is included in SDU's record of processing.

RIO hereby grants permission to commence the processing of the personal data concerned.

In connection with this permit, you must be aware that certain requirements are set for projects involving the processing of personal data. The document "Requirements for processing personal data in research projects" can be downloaded on SDU's website.

Changes must be notified to SDU RIO
If there are any changes in the circumstances that you have stated in the notification, this must be reported to sdu.persondata@sdu.dk, as soon as possible, using SDU's form.

Significant changes require prior authorisation, while minor changes only need to be reported to RIO within 4 weeks of the change. If you are in doubt as to whether the change to your project is significant or minor, please contact RIO for clarification.

Disclosure/transfer

If you need to disclose data to others, or reuse data, please contact SDU RIO on e-mail sdu.persondata@sdu.dk. For more information, see: https://www.sdu.dk/en/anmeldelse and the related guides and forms.

Best regards,

Legal Services
SDU RIO

University of Southern Denmark
Campusvej 55
5230 Odense M
T +45 65 50 10 00
www.sdu.dk
