# Supplementary material for: The Infant Health Study - Promoting mental health and healthy weight through sensitive parenting to infants with cognitive, emotional, and regulatory vulnerabilities: protocol for a stepped-wedge cluster-randomized trial and a process evaluation within municipality settings
Source: BMC Public Health. 2022 Jan 28;22:194. doi: 10.1186/s12889-022-12551-z (PMC8796192; doi:10.1186/s12889-022-12551-z)
Supplement: Supplementary file 4 — Additional file 4. [file 12889_2022_12551_MOESM4_ESM.pdf]

How to participate?

- You will receive a letter via e-Boks with a questionnaire about your child's development and your experiences as parents.
- Only one parent can fill in the questionnaire.
- It takes approximately 15 minutes to fill in the questionnaire.
- Once you have answered the questionnaire, you are included as participants in the project.
- Your health nurse will help you fill in the questionnaire if needed.
- You start on VIPP-PUF and make agreements with the health nurse on a total of 6 visits over approximately 3 months.
- When your child is 18 and 24 months old, we will ask you again to answer a questionnaire.
- When your child is 24 months old, you will have a visit from the health nurse.
- A few parents will also be interviewed, and we will ask you about your child's development and your experiences as

Best regards,

Anne Mette Skovgaard, Professor DM Sci, and
Janni Ammitzbøll, health nurse MHP PhD.

SDU
NATIONAL INSTITUTE OF PUBLIC HEALTH

INFANT HEALTH

Dear parents,

Would you like to help us gain more knowledge about how we support infants' development?

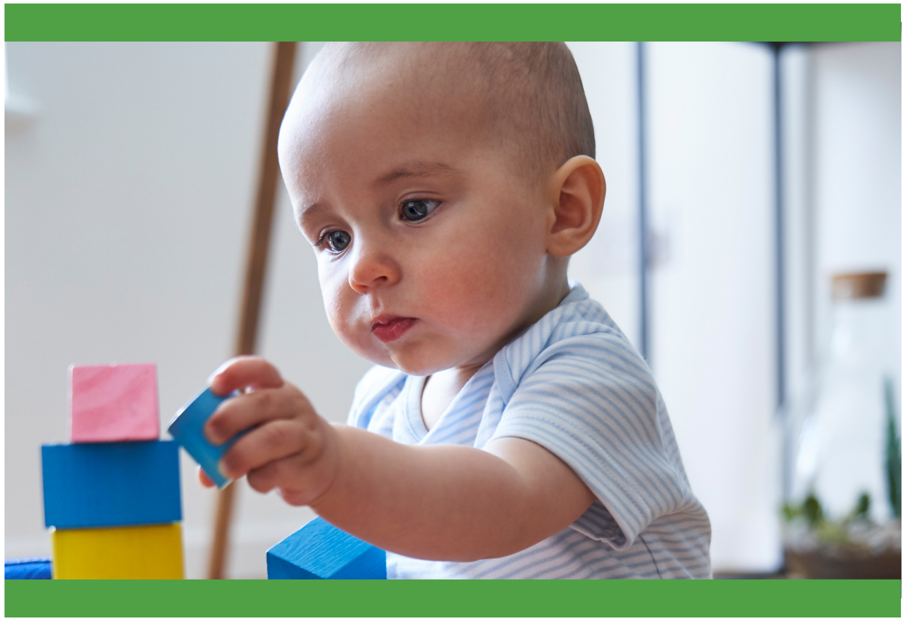

The Infant Health research project
The National Institute of Public Health
University of Southern Denmark

Contact: smaaboern@sdu.dk.

Infant Health

Infant Health is a research project that aims to provide new knowledge about how to prevent infants from developing mental health problems and overweight.

We know today that every sixth child under the age of two has a developmental vulnerability that affects their abilities to develop, play and learn and get a healthy weight development.

Health nurses are in the best position to prevent mental health problems and overweight in early childhood. But we lack knowledge about the best way for the visiting nurse to help children and parents during this period of development.

Therefore, health nurses in a number of Danish municipalities and researchers of infants have developed a new method, VIPP-PUF, which aims to help parents support their child's development.

We need your help to find out whether the new method is better at preventing mental health problems and overweight compared to what health nurses usually do. You will help us by answering questionnaires about your child's development and your experiences as parents when your child is approximately 10, 18 and 24 months old.

In addition, the health nurse will make 6 home visits during approximately 3 months to complete a VIPP-PUF course. During the visits, the health nurse will make small video recordings of your child playing and dining with one parent. The video recordings will form the basis for the visiting nurse's guidance on how to understand and handle your child's signals and reactions.

You can read more about the project at:
www.sdu.dk/da/sif/forskning/projekter/smaa_boerns_sundhed

Who can participate?

During the home visit at 9-10 months, the visiting nurse will assess who can participate in the project. Participating in the project is voluntary, and you can withdraw at any time without it affecting your healthcare provisions.

What is to gain from participating?

- You will receive a gift voucher when you have answered the questionnaire to thank you for your participation.
- Through the VIPP-PUF course, you will get a unique insight into your child's development.
- After the end of the VIPP-PUF course, you will receive a small book that illustrates your child's development with text and pictures.
- You will help research to gain new knowledge about how infants develop and how we can prevent some children from developing mental health problems and overweight.
- You will help the health nurses to become even better at their work and get even better and more targeted initiatives for infants and their parents.

Infant Health is a collaboration between the municipality healthcare and a number of other municipalities and researchers from the National Institute of Public Health, the University of Southern Denmark (SDU) and from the University of Amsterdam, the Netherlands.

The project is approved by the SDU Research & Innovation Organisation (RIO) and the Research Ethics Committee (REC), SDU.

The project is supported by the Independent Research Fund Denmark and the Novo Nordisk Foundation.
