# Supplementary material for: The Infant Health Study - Promoting mental health and healthy weight through sensitive parenting to infants with cognitive, emotional, and regulatory vulnerabilities: protocol for a stepped-wedge cluster-randomized trial and a process evaluation within municipality settings
Source: BMC Public Health. 2022 Jan 28;22:194. doi: 10.1186/s12889-022-12551-z (PMC8796192; doi:10.1186/s12889-022-12551-z)
Supplement: Supplementary file 5 — Additional file 5. [file 12889_2022_12551_MOESM5_ESM.pdf]

SDU
STATENS INSTITUT FOR FOLKESUNDHED

INFANT HEALTH

The Infant Health research project: Processing of your personal information

Dear parent,

Below you will find a description of the formal basis for processing personal information in connection with the Infant Health research project.

The project belongs to the University of Southern Denmark (SDU), which is responsible for the protection of personal information for use in research projects in accordance with current data legislation.

Participating in the project is voluntary. Once you have agreed to participate, your data will be included in analyses to shed light on young children's development. Data is processed anonymously so that it will not be possible to identify you and your child as individuals. You can withdraw from the project at any time, and your participation will not affect any healthcare provisions for you and your child.

The purpose of including personal information in the Infant Health project
The project will provide new knowledge about how we can prevent young children from developing mental health problems and overweight (you can read more at www.sdu.dk/da/sif/forskning/projekter/smaa_boerns_sundhed). In this connection, we need your help to make us more aware of how young children develop. You can help us by filling in an electronic questionnaire when your child is approximately 10, 18 and 24 months old. In order to invite you to participate, we need to be able to send a personal link to the questionnaire to your e-Boks. In this connection, the healthcare services will send your contact information to us. As thanks for your participation, you will receive a gift voucher, when you have answered the questionnaire. Please note that SDU has an obligation to report to the Danish Tax Agency regarding handing out gift vouchers.

<u>The personal information processed is:</u>

The child: Name, civil registration number, health information and information about the child's development as well as physical and mental well-being.

Parents: Name, civil registration number, mobile phone number and possibly residence address and email address, experiences in relation to being parents as well as national register data about health, origin, education, and social and financial conditions.

How we use your personal information

- All personal information is kept confidential and in accordance with applicable legislation.
- The information will only be used for research.
- All communication of research results will take place in anonymised form.
- Data is stored securely in accordance with the data legislation, and only researchers associated with the Infant Health project will have access to data.
- The information will be deleted or fully anonymised no later than five years after the end of the project, which is 31 January 2030.

What does legislation say?
According to the legislation (Section 10 of the Data Protection Act and Article 6(1)(e) of the General Data Protection Regulation), it is not necessary to ask participants for consent to collect and use personal information in a research project which, like Infant Health, is anchored in the municipal healthcare programmes.

This means that legal provisions allow us to use your personal information for research without your consent, but at the same time, legal provisions are your guarantee that the information is only used for research. Therefore, you do not risk that your information will be used for other purposes.

Section 10 of the Data Protection Act and Article 6(1)(e) of the General Data Protection Regulation also provide an opportunity for us to disclose data to other research projects concerning children's development and health.

Your participation in the Infant Health research project
You agree to participate in the project by filling in the electronic questionnaire, to which we will send you a personal link in your e-Boks. When you agree to participate, it means that we collect the information mentioned above and use it in the research project.

If at any time in the process you no longer want to participate, you can withdraw from the project. As a result, we will no longer collect new information about you and your child, but we still have the right to use the information that we have already received.

Additional information
If you have questions about the study, please contact the researchers behind the project at any time by writing to Research Assistant, Ida Voss, at smaaboern@sdu.dk.

If you have questions about data protection and your rights, please contact our data protection officer, Simon Kamber, at dpo@sdu.dk.

If you want to complain about the processing of personal information, please contact the Danish Data Protection Agency via www.datatilsynet.dk.
