# Supplementary material for: The Infant Health Study - Promoting mental health and healthy weight through sensitive parenting to infants with cognitive, emotional, and regulatory vulnerabilities: protocol for a stepped-wedge cluster-randomized trial and a process evaluation within municipality settings
Source: BMC Public Health. 2022 Jan 28;22:194. doi: 10.1186/s12889-022-12551-z (PMC8796192; doi:10.1186/s12889-022-12551-z)
Supplement: Supplementary file 6 — Additional file 6. [file 12889_2022_12551_MOESM6_ESM.pdf]

Review nr. 1

Review of ‘Infant mental health: Supporting infants’ mental health and healthy weight development through community health nurses’ promoting sensitive parenting’

The aim of this original and well-designed study is to implement and evaluate a parent training program that enhances positive, sensitive parenting and promotes child mental health and physical development, in particular counteracting overweight and obesity. The parent training with video-feedback will be introduced into families with high risk of problems with mental and physical health for whom the care as usual does not seem to be sufficiently effective. The research team has developed a basic Psykisk Udvikling og Funktion program in which community health nurses are educated to stimulate children’s health. But the nurses feel they need better tools to address the needs of the most deprived and at risk families to keep the children’s mental and physical health safe and sound. The proposed study adds a video-feedback intervention to care as usual by the community health nurses, which can be delivered by trained nurses in the context of their daily work with families in which an infant is born.

Overall objective and success criteria of the project.

The overall objective is to show that a parent training module can be successfully implemented in the natural setting of community health nurses working with the most deprived families with elevated risks for children’s negative mental and physical health outcomes. The most important environment of infants in the first few years of life is the family, in particular the parents, and if their sensitive responsive behavior can be optimized by video-feedback training a broad array of positive developmental consequences are expected to emerge. Overweight and obesity are increasingly present in ever earlier stages of life and they have far-reaching consequences later in life, for example in elevating the chances of cardiovascular diseases and increased mortality. In infancy and early childhood mental and physical health development are intertwined and stimulated or derailed by similar parenting interactive behaviors. Therefore, both mental and physical health are targeted in the proposed intervention study, and success criteria of the project are related to this aim: elevated sensitive responsiveness of parents, lower percentages of children at 2 years of age who display problem behaviors, as well as lower percentages of children showing overweight or obesity at posttests.

Originality of the proposed project.

The proposed project is highly original for two reasons. First, the proposed study will be one of its kind in implementing the video-feedback parent training in the practice of well-baby clinics and in the daily activities of the community health nurses. They will be empowered to help families in the most deprived settings and at risk conditions to prevent ill health of the children involved. Second, the design of the study is a highly innovative randomized controlled step-wedge study of intervention efficacy, to my knowledge

not used previously in RCTs of training parents of infants to enhance their sensitive responsiveness. This design is innovative as it combines a large sample size with promoting adherence to the trial even in those families that will be asked to be part of the control group: in the end all control families will have had the opportunity to receive the intervention because of the step-wedge nature of the design: no family fulfilling the criteria for at risk status will be left behind.

Overall quality and feasibility of the proposed project.

The quality of the proposed project is superb. The stepped wedge cluster randomized design really is most recent state-of-the-art, and will take care of ethical issues (such as withholding promising support or treatments from those families who need it) as well as statistical problems with randomization, implementation and power. The level of reflection on statistical analyses taking into account the clustered nature of intervention and outcomes is wonderful. The project will become a show-case for conducting a randomized trial in real life and at a grand scale, with more than 1,000 families involved across a two year period. Because of the clever design with carefully planned waves of interventions this project becomes surely feasible, if sufficient funding to support training and intervening is available. The workload for the participants seems doable, although one might entertain the possibility to skip the CBCL in favor of keeping the shorter SDQ, or to include only part of the CBCL as the SDQ might not work at an earlier age than 24 months. Planned random missings or selection of specific sub-scales which are covering externalizing problems might also be reflected upon. The planned video-recording of meal-time interactions is simply great because it will make the observation of sensitive parenting much more valid in this somewhat stressful condition, and because it is of course closely related to one of the goals of this project, namely striving to facilitate healthy weight development.

The project goals.

It is a great to see not only global aims of the study but also specific primary and secondary hypotheses. Because of the specificity of the hypotheses, the detailed design, and the explicit statistical approach it might be possible to pre-register the main part of the study protocol as is now required by more and more journals. The Lancet for example already requires pre-registration of trials to be publishable, as it may counteract fishing for false positives.

Is the given timeframe adequate and realistic?

The planning is sufficiently detailed, and the big advantage of the current project is that it can ‘piggy-back’ on previous work within and with the municipalities that already expressed their enthusiasm for the

project. With the more qualitative pilot to precede the main study motivation of all parties involved – parents, nurses, managers, policy-makers- will be optimal and much of the usual problems with participant recruitment will be addressed adequately. The possibility of connecting the data to available nation-wide registries is an exceptional opportunity to widen the scope and impact of the current project, also in the long-term as this might easily be the start of a longitudinal follow-up study.

Significant risks and weaknesses.

This is an excellent proposal with a highly relevant goal, and an exemplary design. If anything my previous comment on the burden of data-collection for the participants might be taken into account. One might entertain the possibility to include the second caregiver/parent in the study to complete some of the self-report outcome assessments, so as to enhance the validity of the resulting markers for health development.

Organization and governance

The professional environment is excellent, as the applicants did manage to include in their team experts from a large variety of disciplines. All bases are covered, from qualitative approaches in the pilot phase, to implementation of the intervention and to conducting rather sophisticated statistical analyses. The applicants are highly skilled and experienced, as are the members of their team.

Budget

It is difficult for me not being Danish to comment on whether the budget is justified and sufficient. Local knowledge about how these types of projects are carried out in Denmark, what amount of money is required for the various members of the team, research assistants, etc., is needed to make an educated guess about the adequacy of the proposed budget. In other countries (UK, NL) in which I have some experience in budgeting this type of work, my experience has been that it just is more expensive than the usual more descriptive, correlational or epidemiological work, so its budget should be measured with a different standard.

Conclusions and recommendations

My overall conclusion is that this is an excellent proposal and that I can recommend its funding without any reservation. I have already described the main strengths and weaknesses of the proposal, including its stepped wedge cluster randomized design, the involvement and education of community health nurses, the video-feedback intervention and observational measures of parenting, and the careful statistical analyses.

The only small worry I have pointed at above is the burden for the participants of some of the self-report behavior problems questionnaires, and I have suggested some ways to amend this issue. The NNF should certainly assign its highest priority to fund this type of work as it nicely fits in its overall mission, and this specific project is absolutely state-of-the-art.

Review nr. 2

Reviewer report for the Novo Nordisk Foundation, December 12th 2019
Project tittle: "Supporting infants' mental health and healthy weight development through community health nurses' promoting sensitive parenting"

Overall objective and success criteria of the project

Given the high prevalence of mental health problems as well as overweight among children and adolescents, innovative methods supporting systematic, early identification and effective interventions targeting both areas are highly needed. Research supports that early cognitive, emotional and behavioural regulation plays a major role in the later development of mental health and weight-regulation, and that parenting style holds a key position.

The study aims to develop and examine the feasibility, fidelity and effectiveness of a community health nurse led intervention called the PUF-VIPP intervention to promote sensitive parenting of infants with cognitive and regulatory problems at ages 9-10 months.
The primary hypothesis is that the intervention will lead to a reduction of mental health problems in infants at 18 and 24 months (primary outcome).
The secondary hypotheses are that the intervention will lead to
a) reduction of cognitive and regulatory problems (secondary outcome)
b) promote healthy weight development (secondary outcome)
c) reduce parents' experiences of stress and promote sensitive parenting and parents' feeling of competence and relatedness (secondary outcome)

The main succescriteria is defined as a reduction in score at the Strengths and Difficulties Questionnaire (SDQ). The applicants expect that the mean SDQ in children with cognitive and regulatory problems at ages 9-10 months (defined as ≥3 problems in the PUF-screening) is 13 and that it will be reduced by the intervention to 11. The power calculation estimates that a sample size of 790 children would be required to detect this effect. The expected number of included children is approximately 1400.
Other succescriterias are the feasibility, fidelity and effectiveness of the intervention. Face valitdity and feasibility will be tested in two strategically sampled municipalities, and results will be used to adjust the final intervention and study set-up. A process evaluation using quantitative and qualitative data is planned in WP4, and aims to document the development of the PUF-VIPP intervention, and to explore and analyze whether the intervention is implemented as intended. The project will assess the acceptability of the intervention among CHNs and parents, and their appreciation of and satisfaction with the intervention programme.
Regarding the promotion of "a healthy weight development" it is not clearly stated how this is defined, and what the succescriteria is. The outcome is BMI z-scores at age 24 months. It is stated that recommendations from the National Board of Health that include guidelines regarding the prevention of overweight are followed by CHNs.

The originality of the proposed project

I consider that the proposed project has a high originality, since it is the first of its kind to explore intervention within municipality settings to target the earliest developmental trajectories of mental health problems and overweight via promoting sensitive parenting of infants of cognitive and regulatory vulnerabilities. Reaching out to a broad population via CHNs and detecting the most vulnerable children is

crucial, and the study design seems suitable to overcome traditional selection bias, that frequently leaves out the most vulnerable groups.

Combing the already implemented basic programme PUF (in Danish Psykisk Udvikling og Funktion) to identify infants' with cognitive and regulatory problems, with the Video-based Intervention to Promote Positive Parenting (VIPP), is original. The VIPP is validated as a cost-effective tool to promote parental sensitivity and child attachment in vulnerable infants. Potentially this could improve infants health care with a evidence based and systematic approach to support beneficial parenting styles early in life.

The overall quality and feasibility of the proposed project.

Project goals

Main project goals are addressed in the application and includes development of the intervention as well as evaluation of feasibility, fidelity and effectiveness. Project goals are defined in four work packages (WP 1-4).

WP1 describes the project planning and management. Importantly, HCNs from participating municipalities are part of this WP, since they are key stakeholders in the process. The overall goal of developing an invention that adds on to the PUF-programme and addresses sensitive parenting to vulnerable infants by the use of the VIPP seems clear.

In WP2 three main goals for the intervention are described: 1) The intervention must identify the target population, 2) it must be ensured that the intervention can be delivered by CHNs and that 3) the intervention is feasible within the frames of municipality child health care. All three goals seems clear, except that overweight infants with low problem-score in the PUF-screening will not be identified as vulnerable in the currently described recruitment procedures.

In WP2a a pilot study including two strategically sampled municipalities aims to evaluate the face validity and feasibility of the PUF-VIPP-model, focusing on six relevant areas (feasibility and effectiveness of the CHNs education and training, the recruitment procedures, the function of the web-platform, the practical procedures, the feasibility within the existing routines of CHNs, the parents' motivation, compliance and acceptance of the intervention) . Further in WP2a the goals are to adjust the efficacy study (WP3) and the process evaluation (WP4), and to evaluate the study frame, the delivery tools, and the feasibility of the primary, intermediate and secondary measures. All goals seems clear.

WP3 is the efficacy study, focusing on mental health (WP 3a) and weight (WP 3b). Mental health at 24 month is the primary study outcome, assessed by The Strenghts and Difficulties Questionnaire, SDQ. In addition mental health is assessed by other validated tools, including The Child Behaviour Checklist, and the The Ages and Stages Questionnaire, ASQ -3. In WP 3b, the primary focus is the effect of the intervention on weight expressed as Z-BMI score. Measures of length and weight at birth and from CHNs' measurements at home visits (mean four measurements) will be used. It is not described in details how anthropometry will be assessed (hand-held weight, electronic weight, fixed or flexible length-measurement etc.) and how much the number of measurements in each child can vary, as well as possible reasons for variations. Careful datacollection about the child's feeding habits are crucial to the later interpretation of weight development.

WP 4 is an overall process evaluation and aims to answers the crucial question: *What works for the vulnerable families*? A qualitative approach will be taken in the evaluation using scientific sound methods (participant observations, focus group interviews with relevant parties (parents, CHNs) and individual semistructured interviews with mothers and fathers.

Timeframe
The overall timeframe of the project is 66 months (January 2020 – June 2025), and includes all WPs as well as dissimination. The included Gant-diagram explains in detail the timeframe of each WP, and to my best knowledge the suggested timeframes seems adequate and realistic for each WP. There might be a critical overlap in WP2a in the testing and finalizing the intermediate and follow-up questionnaires and home visit assessment, since it takes place at the same time as the intervention starts in Cluster I (WP3, study months 17-19). However, this might not be a problem since the first intermediate follow-up assessment is planned to start in study month 21, thereby leaving adequate time.

Significant risks and weaknesses associated with the chosen approach described in the application.
Risks: Involving 14-17 municipalities implies the risk that the economy and priorities may change over a 3-5 year period in each municipality. However, the project builds on already well-established collaboration and a successfully implemented model (the PUF-model), thereby increasing the likelihood that municipalities will not drop-out during the study. The timeframe will be vulnerable to staff-absence and all periods overlapping public holidays/vacation are known to be sensitive to delays. This may be taken into account in any revision of the timeline.
The continuous needs for educating and sustaining staff skills in VIPP-method might be a challenge during the study, since CHNs change jobs, go on maternity leave etc.
In addition working with vulnerable families often implies no-shows and drop-outs. However, the powercalcualtion shows that power should be sufficient to meet a substantial drop-out, should it happen.

Weakness: The proposed design will not necessarily identify children with an unhealthy weight development, if the sole criteria for inclusion is PUF-score >3. This might be justified, if you accept the premise that regulatory difficulties precede disturbed eating, leading to overweight.

Organization and governance
The professional environment.
The PI is in an expert in the research field of child and adolescent psychiatry, and has studied the of development of mental health problems in childhood (epidemiology of developmental psychopathology, regulatory disorders in infants and young children, problematic eating behavior and eating disorders in preschool age, mental symptoms of somatic diseases) throughout her scientific career. She has led the largest study in the field since 2000 (CCC 2000) and has extensive research management experience.

The co-PI is a master's in public health, with relevant scientific background and specific research experience in the field. Her experience in supervision and research management is mainly based on supervising bachelor-students.

The other involved researchers are very strong in the field of epidemiology and methodology, development and evaluation of methods, childhood growth and qualitative research. Overall the project has a strong scientific profile with robust collaborations with national and international experts in the field.

In addition, there is an ongoing collaboration with the involved municipalities and the CHNs, which is considered a major strength in the professional environment, and I believe that the involved partners have the skills and resources that are needed for the project.

Budget
The total budget is DKK 25.160.568 and includes WP 1-4 and an overhead of 5%. The budget seems realistic and covers the planned activities. Two PhD students are expected to graduate from the project. No supervision of PhD students is in the budget and might be considered a co-financing. The listed co-financing is DKK 6.020.407.

Conclusions and recommendations

The proposed project “Supporting infants’ mental health and healthy weight development through community health nurses’ promoting sensitive parenting” aims to develop and test an intervention targeted to vulnerable children and their parents to reduce childhood mental problems and prevent overweight. In the light of the morbidity, mortality and costs due to these two major public health problems, this project has the potential to add new knowledge in the field. The described aims, hypothesis and methods are based on previous literature as well as scientific experience embedded in the group of applicants. The project uses scientific sound methods that are expected to give robust results, that can be compared to other studies in future research. The design profits from already well established collaboration with many municipalities, thereby enhancing the feasibility of the study. The project originate from a strong research collaboration with national and international researchers. My main concern is that the main scientific focus is on mental health, and not necessarily on the prevention of overweight. I recommend that NNF consider funding of the proposed project, if the addressed concerns are taken into revision of the proposal.

NNF Review.

INFANT HEALTH: Supporting infants' mental health and healthy weight development through community health nurses' promoting sensitive parenting

• *Please comment on the overall objective and success criteria of the project.*
This is potentially a very valuable contribution to the services offered to children with psychosocial and/or neurodevelopmental vulnerabilities, and consequently to broad public health aims. It addresses two issues of major importance, namely mental wellbeing and obesity, and there is a plausible case that intervention in early childhood is the best way to prevent these problems for many individuals. The study aims to build upon existing services by training staff to offer the VIPP intervention when multiple needs are identified by community health nurses. There is much to be said in favour of this progressive universalist approach to public health interventions. While it is not entirely true that the VIPP intervention is the only candidate intervention to improve the quality of parent-infant interaction (VIG[1] and Mellow Babies[2], for example, are alternative approaches), VIPP has been used in the public health nursing context and it is probably the most promising and realistic intervention for individual families with infants available at present.

• *Please comment on the originality of the proposed project.*
This is a highly original project, made possible by the innovative work which has led to the adoption of the PUF system in 30 municipalities. Consistency in developmental assessments is a prerequisite for generalizable early childhood intervention studies and there are few other environments in which cutting edge work of the type proposed here could be conducted. The VIPP is a very promising intervention programme to enhance parent-child interaction and the collaboration between Bakermans-Kranenburg's group and the Danish applicants appears to offer a unique opportunity to evaluate a targeted infant mental health programme in a public health setting.

• *Please assess the overall quality and feasibility of the proposed project.*
o Are the project goals sufficiently clear?
In my view the project goals are well expressed, though I would have liked to have seen a within-trial health economic evaluation.
o Is the given timeframe adequate and realistic?
In general yes, though there are significant risks of delay associated with recruitment to the trial – see below. The proposed plan for the development and piloting of the VIPP intervention is excellent.

• *Please elaborate on any significant risks and weaknesses associated with the chosen approach described in the application.*
The stepped-wedge design has many advantages in real-world trials of the type described here, particularly when negotiations need to take place with senior management of community services who all wish to have access to a promising intervention. The stepped wedge design gives them all reassurance that the intervention will eventually be accessible. There are however a number of serious drawbacks, and these frequently lead to failure of stepped-wedge trials – they are notoriously difficult to complete successfully. One potential drawback is the potential for policy or economic factors to lead to variations in services available over time. Sometimes these factors can prove fatal to a study, for example if there is a radical service redesign or a national policy directive that impacts upon the service being evaluated.
As with all community-based randomised trials, there is always a risk that recruitment will be substantially slower and/or more difficult than anticipated. There are many possible barriers to recruitment. At a cluster/municipality level, there are significant challenges. The demands on the municipalities are considerable: essentially all participating municipalities have to commit to screening the whole population of eligible children in a consistent way, recruiting children scoring over the agreed threshold to the trial both before and after the 'switch point' and then delivering the intervention to all participating children after the switch. I note that 13 expressions of municipality support are provided but not all have provided a firm commitment to participate. I would suggest that a stop-go point based on real municipality engagement might be a good idea to protect the funder's interests.

There is also a risk that political or economic circumstances (or even a change in senior management) might make it difficult for some municipalities to continue involvement during the life of the study.
At an individual participant level there are also risks relating to recruitment: for example it is possible that fewer families than expected would wish to take up the offer of a video intervention. The applicants estimate that 80% of eligible families will participate: this seems optimistic. Families with significant problems are known to be more difficult to engage in research, and are much more likely than others to drop out of longitudinal studies[3].
More importantly there are risks of differential recruitment to the intervention and control groups. Public health nurses might be more likely to recruit participants if they think they have something useful (eg VIPP) to offer to families, in which case the intervention arm of the study will be over-represented. Alternatively the nurse might feel less able to recruit to the intervention arm because of time constraints, in which case the control arm will be over-represented. In individually randomised trials these issues are not so important (though differential attrition might be) and in conventional parallel cluster trials it is at least relatively easy to identify differential recruitment to the two arms of the trial and take early action to correct any imbalance. This is much more difficult in a stepped-wedge trial and it would be sensible to ensure that expert statistical help is obtained to monitor recruitment rates, perhaps through a data monitoring committee.
Although the authors quote Woertman's paper[4], which states that stepped-wedge designs can increase the power of cluster randomised trials, this consideration may not apply in the present study where new participants are recruited at each time point, as pointed out by Hemming[5], specifically when the intracluster correlation (ICC) is relatively low. I think that some expert statistical advice might be helpful in evaluating the power calculation presented here. It may be that a simple parallel-group cluster RCT might be a more efficient design.

In terms of weaknesses:

- There are some intrinsic weaknesses in the stepped wedge design. Even when there is no major change in the clinical or social environment over time, account needs to be taken of the fact that the world changes for families in many ways, for example in terms of the season or the availability of work – in other words temporal clustering needs to be considered. In this case there are three temporal clusters, recruited at intervals of four months. This means that there will be more participants in the intervention group in the autumn and more in the control group in the spring. It is difficult (as it always is) to estimate an ICC for this sort of factor.
- it would have been valuable to have some health economic element to the evaluation. There is increasing interest in this area, and the SDQ, for example, has been mapped to a range of preference-based outcomes[6], potentially allowing the calculation of a cost-per-Quality-Adjusted Life Year for VIPP in this context. This would help commissioners of services internationally to make decisions about the value of introducing training programmes.
- A health economic evaluation would require some additional data collection – for example the EQ-5D could be completed by parents, along with a service use questionnaire. Additional economic data could be obtained from administrative datasets.
- Some of the proposed baseline and outcome measures could potentially be improved. Parental mental health should be assessed directly, perhaps by the HADS or EPDS. The ASQ3 does not have social and emotional functioning items. The ASQ-SE is more appropriate but I believe the latest version is not yet available in Danish. The ASQ does not have a particularly strong track record as a trial outcome. For social and emotional functioning below two years, the BITSEA[7] might be a better (though imperfect) choice. The CBCL is not well liked by parents in non-specialist settings and I suspect it will be unpopular in this context – the SDQ has good concurrent validity[8] and may be an equally good choice[9].
- A simple measure of expressive language should be added to the outcome measures. Normal language development depends on adequate cognitive abilities and good communication between parent and child. It is a strong predictor of psychopathology. A good tool might be the Danish 100-word Macarthur Communicative Development Inventory[10]
- Parental sensitivity is a key outcome of this study, and it will be evaluated independently using home video recordings. There is no detail about what method is to be used for this measurement, and there are several alternative methods available, none of which is perfect. This is a changing field however, and it may be that useful tools will emerge during the life of the trial. In any event, the Statistical Analysis Plan does not need to be finalised and published until the final dataset is locked so it is not essential to

make a commitment to the video analysis system at this stage. It should be noted however that analysis of video material of this type can be quite expensive.

Organization and governance

• Please evaluate the professional environment.

The research team is strong. Skovgaard has a sound record in delivering high-impact results from community-based studies, while Pontopiddan has broad experience in conducting parenting trials in the Danish context, specifically in collaboration with municipalities. Bakermans-Kranenburg is probably the leading academic internationally working in the field of parental sensitivity and interventions to improve it. Ammitzbøll is one of the few doctoral-level public health nurses with quantitative research expertise. Tolstrup has a broad methodological expertise in public health and epidemiology and Tjørnhøj-Thomsen will provide qualitative expertise of value to the process evaluation. I am less familiar with the other applicants' work but they appear to have complementary skills.

I think that the governance of the project would be strengthened by the appointment of an independent trial steering committee, and possibly an independent data monitoring committee

• Please assess whether the necessary experience and skills or other resources available are sufficient for the proposed project.

In general yes, but I think that a health economist would be a good addition to the team, as well as a biostatistician with complex trial methodology expertise.

Budget

• Please comment on whether the budget is justified and sufficient in light of the proposed project and the project setup.

The budget seems reasonable, but I would recommend a stop-go point as mentioned above to protect the funder in case of poor recruitment of municipalities and/or participating families.

Conclusions and recommendations

• Please provide an overall conclusion and recommendation. What are the main strengths and weaknesses of the application? Please summarize merits and concerns.

This is a highly original study of a promising area for potential public health intervention. The design is made possible by a strong foundation in Danish public health nursing, a well-designed intervention and a good plan for customising the intervention to the Danish Municipality environment. The overall design of the effectiveness study is challenging and it may be that a more conventional cluster RCT could be more efficient, but this is a matter for debate. There is a need for reconsideration of some of the baseline and outcome measures and an economic evaluation should be considered. As always in any public health trial, recruitment issues could prove to be difficult.

• Is there justification for the NNF's possible consideration of funding the proposed project?

Yes, if the comments above are taken into account.

1. Hoffenkamp HN, Tooten A, Hall RAS, et al. Effectiveness of hospital-based video interaction guidance on parental interactive behavior, bonding, and stress after preterm birth: A randomized controlled trial. *Journal of Consulting and Clinical Psychology* 2015;83(2):416-29. doi: 10.1037/a0038401
2. MacBeth A, Law J, McGowan I, et al. Mellow Parenting: systematic review and meta-analysis of an intervention to promote sensitive parenting. *Developmental Medicine & Child Neurology* 2015;57(12):1119-28. doi: 10.1111/dmcn.12864
3. Wolke D, Waylen A, Samara M, et al. Selective drop-out in longitudinal studies and non-biased prediction of behaviour disorders. *British Journal of Psychiatry* 2009;195(3):249-56.

4. Woertman W, de Hoop E, Moerbeek M, et al. Stepped wedge designs could reduce the required sample size in cluster randomized trials. *J Clin Epidemiol* 2013;66(7):752-8. doi: 10.1016/j.jclinepi.2013.01.009 [published Online First: 2013/03/26]
5. Hemming K, Girling A. The efficiency of stepped wedge vs. cluster randomized trials: Stepped wedge studies do not always require a smaller sample size. *Journal of Clinical Epidemiology* 2013;66(12):1427-28. doi: https://doi.org/10.1016/j.jclinepi.2013.07.007
6. Boyer NR, Miller S, Connolly P, et al. Paving the way for the use of the SDQ in economic evaluations of school-based population health interventions: an empirical analysis of the external validity of SDQ mapping algorithms to the CHU9D in an educational setting. *Qual Life Res* 2016;25(4):913-23. doi: 10.1007/s11136-015-1218-x
7. de Wolff MS, Theunissen MH, Vogels AG, et al. Three questionnaires to detect psychosocial problems in toddlers: a comparison of the BITSEA, ASQ:SE, and KIPPPI. *Acad Pediatr* 2013;13(6):587-92. doi: 10.1016/j.acap.2013.07.007 [published Online First: 2013/11/19]
8. Goodman R, Scott S. Comparing the Strengths and Difficulties Questionnaire and the Child Behavior Checklist: Is small beautiful? *Journal of Abnormal Child Psychology* 1999;27(1):17-24.
9. Warnick EM, Bracken MB, Kasl S. Screening Efficiency of the Child Behavior Checklist and Strengths and Difficulties Questionnaire: A Systematic Review. *Child and Adolescent Mental Health* 2008;13(3):140-47.
10. Vach W, Bleses D, Jorgensen R. Construction of a Danish CDI short form for language screening at the age of 36 months: methodological considerations and results. *Clin Linguist Phon* 2010;24(8):602-21. doi: 10.3109/02699201003710606

Review nr. 4

Peer review of 'INFANT HEALTH: Supporting infants' mental health and healthy weight development through community health nurses' promoting sensitive parenting' for NFF

Please comment on the overall objective and success criteria of the project.

This project addresses an important and timely issue in its focus on infant mental health and overweight. The specific objective regarding development of the intervention tool and assessment of feasibility is appropriate to the current state of the evidence. The incorporation of a pilot study is important in this regard, although it does seem to be assumed that the outcomes of the pilot will be sufficiently positive to warrant moving directly to the full trial (see further comment on this below).

Please comment on the originality of the proposed project.

The project is one of those that seems like such an obvious next step in the field as to appear initially to be self-evident rather than original. But there is no other work like this – not only screening for cognitive and regulatory vulnerabilities in the first year of life, but doing so on a whole population basis, and then going on not only to provide an intervention in a community setting, but also combining the focus on infant mental health and overweight is internationally unique. It is highly novel partly because there are very few places in the world with the infrastructure to allow such a project. This project will make an important novel contribution to the field, potentially opening up research on comorbid mental and physical health problems.

Please assess the overall quality and feasibility of the proposed project. Are the project goals sufficiently clear?

Yes, the goals are clear. The narrative of the proposal combined with the graphical elements make the goals at each stage, as well as the overall goal, clear. o Is the given timeframe adequate and realistic? The project is trying to achieve a lot, but is built on a sound foundation and there is good reason to believe it will be feasible to conduct the study as planned.

Please elaborate on any significant risks and weaknesses associated with the chosen approach described in the application.

In general, this is a well-planned project based on a strong foundation. One of its key strengths is the almost whole population uptake of the child health surveillance system in Denmark. Although results thus far have shown good uptake of the PUF infant mental health screening, I wonder if the same level of participation will be achieved in a trial? Given that there is a strong focus on ensuring the most vulnerable families are included, do the applicants have evidence that this will be achievable? Another concern is surrounding the development of the new intervention – if I have understood correctly, the intention is to essentially combine two existing approaches (PUF-programme and VIPP) and to add in a component that directly addresses issues around overweight. The aim is to deliver all of this within six sessions without diluting any of the effective components, which may be ambitious. I do not wish to sound sceptical, more that I think careful monitoring of this during the pilot stage will be necessary. I wonder as well about the potential for including a stop/go point of sorts after the pilot stage. The application has been written with

an optimistic approach assuming that the outcomes of the pilot will justify continuation to a full trial. Perhaps the applicants should consider what criteria might cause them to decide to halt the research at this stage, rather than to proceed? There is reference made in the proposal to the design of the new intervention taking into account '…the traditions and strategies of the particular municipality…' (p13) and later that it will '…take into account the conditions and needs of the individual families…' (p15). This suggests a relatively flexible approach to the intervention, which is a good thing for complex interventions such as this, but also presents a tension with the rigidity of the RCT method (i.e., can we be sure that all participants received a sufficiently similar intervention?). I would hope to see the process evaluation closely monitor how this flexibility plays out in reality, and the extent to which the researchers will be able to clearly define 'PUF-VIPP' both within this research and for future application. Finally on the methods, I am curious as to which method of coding will be used in the video observations, as well as what sort of 'unspecific measures' of infant development are used by the CHNs – an example would be good. Being unspecific about measures leads to concern about the replicability of the work.

Organization and governance

Please evaluate the professional environment.

The proposed study will take place in a very strong professional environment. As well as the skills and experience of the research team itself (see comments below) the existing working relationship between the university researchers and the municipalities / health service nurses is the sort of collaboration that can take a lot of time and energy to establish. The fact that this exists and has already produced good quality research outputs is a clear advantage of this proposal.

I am left a little uncertain as to the management structure for the project, however. In the narrative part of the application, there is reference to the research group, the Project Steering Group, and the Participatory planning group. However, in the Gantt chart (Appendix 2) I see reference to a 'Scientific steering committee' and an 'advisory board'. It is not clear how these two sets of groups relate to each other. I would like to have seen not only clarity on this issue, but also more information about the day-to-day management and environment of the project, as most of my positive recommendation in this respect has to be based on reputation rather than articulated structure / process. The current articulation suggests this has been something of an afterthought on the part of the applicants. For a study of this size I would advise the inclusion of an independently-chaired Trial Steering Committee and possibly a Data Monitoring Committee. There should at very least be one independently-chaired advisory group that can make key decisions about the trial.

Please assess whether the necessary experience and skills or other resources available are sufficient for the proposed project

There is a well-qualified interdisciplinary team with a breadth and depth of relevant experience to make this project a success. The preceding research, the resultant publications, as well as the statements of collaboration are all testament to this (although I could not read those in Danish and it was not possible to copy and paste into a translation website). The involvement of Marian Bakermans-Kranenburg and Maiken Pontoppidan in particular is a positive addition beyond the existing research team.

Budget

Please comment on whether the budget is justified and sufficient in light of the proposed project and the project setup.

I find it difficult to comment fully on this as a non-Danish researcher (with a different currency and cost of living to contend with). I also do not entirely follow the provided spreadsheet – I had assumed that the 'cofinancing' section refers to existing funding not being requested of NFF (as alluded to in the application text), but on review I am not sure this can be true. If my assumption is correct, then the existing co-funding does mean that this study presents very good value for money. Looking at the budget headings, it appears that the applicants have thoroughly considered the costs of the project in a realistic way, and have ensured it will be appropriately staffed, including support staff which is so often under-costed.

Conclusions and recommendations

Please provide an overall conclusion and recommendation. What are the main strengths and weaknesses of the application? Please summarize merits and concerns.

Overall this is a strong application for an important piece of work to be conducted by a world-leading team well-placed to make this project a success. The objective of the work is timely and well-grounded in the existing evidence. It is also ground-breaking as there is little to no evidence of effective screening or intervention of this type in families of such young children. The application is strong in its articulation of the basis for the research, its foundation in the existing PUF-programme research, consideration of the stages required to establish the intervention and rigorously test its effectiveness, and the need to adopt a multidisciplinary approach. The application is weaker in its articulation of the operationalisation of the project – its management structure, timing of meetings, justification of budget (there was none), and of the timeline (the Gantt chart is very good, but some narrative explanation is required). I would have preferred to have seen more of this level of detail, as well as consideration of the opportunity to pause the project after the pilot, should the results suggest a full trial would not be warranted or feasible.

Is there justification for the NNF's possible consideration of funding the proposed project?

I would say it is entirely justified to consider this project for funding. Its weaknesses are either minor or easy to remedy (and given the pedigree of the research team entirely achievable). I would have no hesitation in supporting the funding of this project.
